# Developmental auditory deprivation in one ear impairs brainstem binaural processing and reduces spatial hearing acuity

**DOI:** 10.1371/journal.pbio.3003337

**Published:** 2025-09-05

**Authors:** Kelsey L. Anbuhl, Alexander T. Ferber, Andrew D. Brown, Victor Benichoux, Nathaniel T. Greene, Daniel J. Tollin

**Affiliations:** 1 Department of Biomedical Sciences, Creighton University School of Medicine, Omaha, Nebraska, United States of America; 2 Center for Neural Science, New York University, New York, New York, United States of America; 3 Neuroscience Training Program, University of Colorado Anschutz Medical Campus, Aurora, Colorado, United States of America; 4 Department of Physiology and Biophysics, University of Colorado Anschutz Medical Campus, Aurora, Colorado, United States of America; 5 Medical Scientist Training Program, University of Colorado Anschutz Medical Campus, Aurora, Colorado, United States of America; 6 Department of Speech and Hearing Sciences, University of Washington, Seattle, Washington, United States of America; 7 Department of Otolaryngology, University of Colorado School of Medicine, Aurora, Colorado, United States of America; 8 Speech, Language, and Hearing Sciences, University of Colorado Boulder, Boulder, Colorado, United States of America; University College London, UNITED KINGDOM OF GREAT BRITAIN AND NORTHERN IRELAND

## Abstract

Early sensory experience can exert lasting perceptual consequences. For example, a brief period of auditory deprivation early in life can lead to persistent spatial hearing deficits. Some forms of hearing loss (i.e., conductive; CHL) can distort acoustical cues needed for spatial hearing, which depend on inputs from both ears. We hypothesize that asymmetric acoustic input during development disrupts auditory circuits that integrate binaural information. Here, we identify prolonged maturation of the binaural auditory brainstem in the guinea pig by tracking auditory evoked potentials across development. Using this age range, we induce a reversible unilateral CHL and ask whether behavioral and neural maturation are disrupted. We find that developmental CHL is associated with alterations in a brainstem readout of binaural function, an effect that was not observed in a separate cohort with adult-onset CHL. Startle-based behavioral measures suggest that Early CHL animals exhibit reduced spatial resolution for high-frequency sound sources. Finally, single-unit recordings of auditory midbrain neurons reveal significantly poorer neural acuity to a sound location cue that largely depends on high-frequency sounds. Thus, these findings show that unilateral deprivation can disrupt developing auditory circuits that integrate binaural information and may give rise to lingering spatial hearing deficits.

## Abstract 2

Early unilateral auditory deprivation can cause lasting spatial hearing deficits. This study shows that unilateral hearing loss during development impairs binaural brainstem function and spatial hearing acuity in guinea pigs, effects not observed with adult-onset hearing loss.

## Introduction

Unilateral sensory deprivation during development can profoundly alter neural circuits that rely on bilateral integration. Classically, monocular visual deprivation or eyelid closure during discrete developmental periods induces structural and functional changes in primary visual cortex neurons [1–7] and leads to deficits in visually-guided behaviors requiring binocular perception [8–11]. Similarly, unilateral manipulations in the somatosensory system (e.g., whisker trimming) disrupt dendritic morphology, synaptic physiology, and neural responses in the barrel cortex [12–17]. In the auditory system, unilateral deprivation alters binaural selectivity in the auditory cortex [18–22], thalamus [23], and midbrain [22,24–27], and can lead to binaural and spatial hearing deficits [19,20,28]. While plasticity has been demonstrated in the cochlear nucleus of adult animals following a unilateral hearing loss [29], it remains unclear whether unilateral deprivation also induces plasticity in downstream nuclei of the auditory brainstem, where binaural integration first occurs [30,31], and how such changes might affect spatial hearing.

The superior olivary complex in the auditory brainstem contains neural circuits specialized to extract subtle differences between the times of arrival (interaural time difference, ITDs) and amplitudes (interaural level difference, ILDs) of sound stimuli at both ears [32,33]. The binaural cues to sound location, ITDs and ILDs, allow for the localization of sound in the horizontal plane and are initially computed at the medial and lateral superior olive (MSO, LSO), respectively. Neurons in the LSO encode ILDs and onset ITDs by integrating excitatory, glutamatergic inputs from the ipsilateral cochlear nucleus with inhibitory, glycinergic inputs from the contralateral ear via large synapses from the medial nucleus of the trapezoid body (MNTB) [33,34]. After hearing onset, the superior olivary nuclei display significant synaptic reorganization of tonotopic maps which is not observed in the cochlear nucleus, a monaural structure [30]. This refinement of tonotopy is likely to reflect experience-dependent alignment of frequency-specific inputs from each ear, which is evident in the pruning of MNTB axon terminals and LSO dendrites and the improvement of frequency selectivity in developing gerbils after hearing onset [35]. This suggests that binaural circuits might be vulnerable to asymmetrical hearing loss, especially considering neurons in the LSO integrate ipsilateral and contralateral afferents that need to be temporally and spectrally matched to maintain precision in ILD and ITD encoding. Indeed, unilateral deafferentation of the cochlea disrupts specificity of MNTB arborizations [36].

Children who experience conductive hearing loss (CHL) due to otitis media with effusion (i.e., accumulation of fluid in the middle ear) often display binaural hearing impairments that persist months to years after resolution of the hearing loss and restoration of normal audibility [37–43]. A physiologically-derived biomarker for binaural function − the binaural interaction component (BIC) of the auditory brainstem response (ABR) − has been shown to be significantly altered in children with a history of CHL [44–47]. The ABR is an auditory evoked potential that comprises synchronous activity of each locus of the ascending auditory system, including the auditory brainstem. The binaural component (i.e., BIC) can be derived from the ABR and has origins in the LSO [48–51]. This raises the possibility that early hearing loss can induce plasticity in the auditory brainstem.

To address this question, we first characterized the development of binaural function in a precocial rodent, the guinea pig, and examined whether unilateral hearing loss during this developmental period disrupts the maturation of neural tuning to binaural cues in the brainstem and midbrain. We found that a brainstem readout of binaural function (i.e., BIC of the ABR) displays a prolonged maturation period suggesting that binaural plasticity may be heightened during this time, and therefore vulnerable to auditory experience. Indeed, we found that a reversible unilateral hearing loss in development disrupts the BIC of the ABR, whereas hearing loss of the same duration induced in adult animals did not give rise to BIC impairments. We then asked whether Early CHL also contributes to spatial hearing deficits. We found that Early CHL animals displayed larger minimum audible angles (MAAs) for high-pass, but not broadband, noise stimuli compared to Control animals. Finally, in vivo single-unit recordings of auditory midbrain neurons revealed diminished neural acuity to interaural level difference (ILD) cues to sound location. Taken together, these results suggest that asymmetric auditory input during development can disrupt binaural circuits at the level of brainstem and midbrain that support spatial hearing, and may, in part, explain the spatial hearing deficits observed in animals and children following developmental CHL.

## Results

### Prolonged maturation of binaural auditory brainstem function

**Fig 1:**
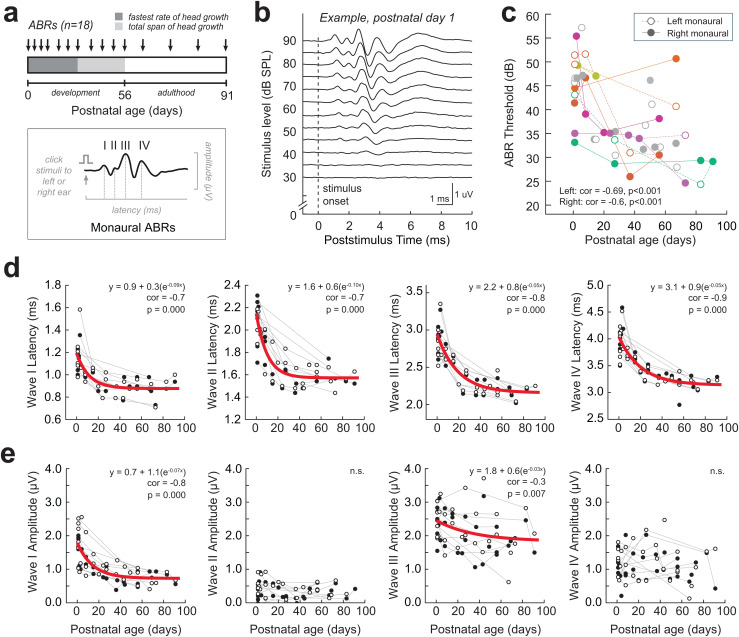
Development of guinea pig monaural auditory brainstem responses (ABRs). (a) *Inset*: The ABR is an auditory-evoked potential that displays peaks (black trace, waves I–IV) corresponding to locations along the ascending auditory pathway. Presentation of click stimuli to the left or right ear alone elicits a monaural ABR. *Timeline*: ABRs were collected in guinea pigs (*n* = 18) at developmental ages spanning newborn (Postnatal day 1; P1) through adulthood (>P56). A subset of guinea pigs (*n* = 7) had ABR measurements tracked across development, and the remaining animals (*n* = 11) had 1−2 ABRs at different developmental ages. (b) Example monaural ABR trace (right ear) for subject ID #124111 at P1. Broadband click stimuli are presented across a range of stimulus intensities (30−90 dB SPL in 5 dB steps). Traces shown are the average of 500 repetitions. The dotted vertical line indicates the click stimulus onset. ABR threshold is defined as the lowest intensity click that elicits a clearly detectable ABR waveform (e.g., threshold in this example is 40 dB SPL). (c) Click-ABR thresholds as a function of developmental age (P1–P90). Circles indicate individual thresholds from the left (open circles) and right ear (filled circles) ABR measurements. Lines connect data from the same animal tracked over time. Click-ABR thresholds decrease significantly with age (Left correlation = −0.69, *p* < 0.001; Right correlation: −0.6, *p* < 0.001). (d, e) Monaural ABR wave I–IV latency **(d)** and amplitude **(e)** plotted as a function of age for 80 dB SPL monaural clicks. Each column indicates a different wave (I–IV). Circles indicate values from the left (open circle) or right ear (filled circle) monaural ABRs. Gray lines connect data points from the same animal tracked over time. When a significant correlation is found, an exponential decay function is fit to the data (red line). Significant correlations are found for wave I–IV latency (*p* = 0.000 for all waves) and waves I and III amplitude (*p* = 0.000–0.007). The data underlying this figure can be found at https://doi.org/10.17605/OSF.IO/KAW34.

First, we sought to characterize the developmental time-course of binaural brainstem function and subsequently, how asymmetric perturbations of acoustic experience can alter this maturation. Studies examining binaural physiology have often used invasive techniques that prohibit the tracking of binaural neuron function within the same animal over time [34,35,52]. To circumvent these limitations, we used the auditory brainstem response (ABR) as a noninvasive measure of brainstem function. In the guinea pig, there are four to five distinct ABR waves (Fig 1A), each originating from synchronous activation of neural structures along the ascending auditory pathway [31,53–56]. The first three waves are monaural, reflecting activity from a single ear input. Wave I originates from the auditory nerve, Wave II by the cochlear nucleus, and Wave III is thought to arise from the medial nucleus of the trapezoid body [47]. Waves IV and V are associated with activation of the superior olivary complex, the first site of binaural convergence from both ears, and the lateral lemniscal tract, which relays information to the auditory midbrain (i.e., inferior colliculus). Here, we tracked ABR development in the guinea pig, a precocial mammal that shares similar auditory sensitivity as humans [57,58]. Since the acoustical properties of the developing guinea pig stabilize and reach adult-like ranges around postnatal (P) day 56 [59], we collected ABRs from birth (P1) through ages well beyond P56 (>P91) in a group of guinea pigs (*n* = 18).

We first characterized the maturation of monaurally-evoked ABRs. Fig 1B shows an example ABR from a newborn guinea pig (P1), where monaural clicks (i.e., presented to one ear alone) were presented across a range of sound intensities (30−90 dB SPL). ABR thresholds were defined as the lowest intensity that elicits a reliable ABR waveform. We find that monaural ABR thresholds decreased significantly with age (*Spearman’s rho correlation*: Left monaural, *r*_*s*_ = −0.71, *n* = 38, *p* = 0.000; Right monaural, *r*_*s*_ = −0.54, *n* = 38, *p* = 0.002), with the greatest decrease occurring in the first 1−2 postnatal weeks (Fig 1C). We then examined individual ABR waves for developmental changes in wave amplitude and latency (assessed at 80 dB SPL), which are reflective of synchronous sound-evoked neural activity and timing of the ascending auditory nuclei, respectively. We find that the amplitudes of monaural waves I and III significantly decrease with age (wave I: *r*_*s*_ = −0.75, *p* = 0.000; wave II: *r*_*s*_ = −0.16, *p* = 0.223; wave III: *r*_*s*_ = −0.34, *p* = 0.007; wave IV: *r*_*s*_ = −0.13, *p* = 0.331), along with a significant decrease in latencies of all monaural waves (wave I: *r*_*s*_ = −0.72, *p* = 0.000; wave II: *r*_*s*_ = −0.73, *p* = 0.000; wave III: *r*_*s*_ = −0.83, *p* = 0.000; wave IV: *r*_*s*_ = −0.85, *p* = 0.000; Fig 1D and 1E). In general, the latency of the earlier waves stabilized by approximately P21 and the later waves stabilized by approximately P56, suggesting that more peripheral monaural auditory structures (i.e., auditory nerve, cochlear nucleus) mature earlier in postnatal development than later peaks associated with superior olivary complex nuclei.

**Fig 2:**
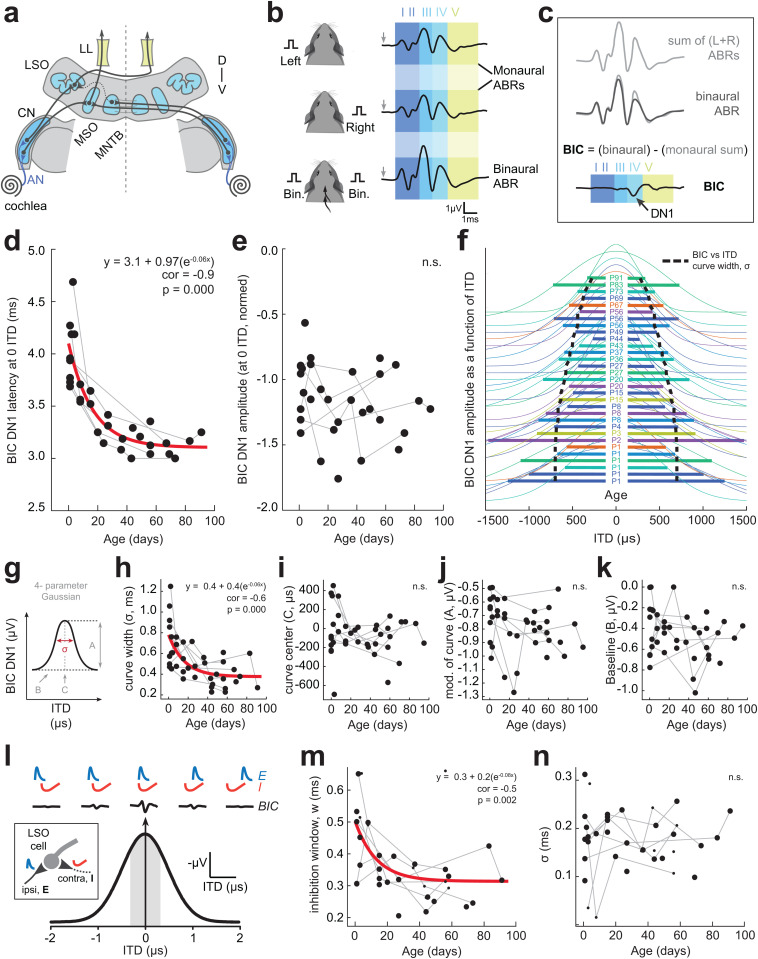
Development of binaural auditory brainstem physiology. (a) Cross-section through the auditory brainstem and the neural generators of the auditory brainstem response (ABR): AN = auditory nerve, CN = cochlear nucleus, LL = lateral lemniscus, LSO = lateral superior olive, MSO = medial superior olive, MNTB = medial nucleus of the trapezoid body. D = dorsal, V = ventral. (b) Presentation of click stimuli to the left ear or right ear alone elicits a monaural ABR (black traces, top). Presentation of click stimuli to both ears elicits a binaural ABR (black trace, bottom). (c) When the left and right monaural ABRs are summed (light grey) and compared to the binaural ABR (dark grey), there is a difference in potentials in ABR wave IV, which corresponds to the superior olivary complex (i.e., LSO, MSO) a location that integrates binaural information. The difference in binaural and the summed monaural potential gives rise to the binaural interaction component (BIC), which contains a negative DN1 peak (arrow). (d) ABRs were collected in guinea pigs (*n* = 18) at developmental ages spanning newborn (P1) through adulthood (>P56). A subset of guinea pigs (*n* = 7) had ABR measurements tracked across development, and the remaining animals (*n* = 11) had 1−2 ABRs at different developmental ages. The BIC was computed for each ABR assessment. BIC DN1 latency at 0 µs ITD decreased significantly with age, as confirmed by Spearman’s rho (*p* = 0.000); an exponential decay function was fit to the data (red line). (e) Normalized amplitude of the BIC DN1 peak at 0 µs ITD was not significantly affected by age (Spearman’s rho, *p* = 0.054). Gray lines: data points from the same animal tracked over time. (f) BIC DN1 amplitude as a function of ITD (fitted with a Gaussian model; centered and normalized) for each ABR time point across development. Colors indicate each animal tested. Horizontal lines indicate the curve width (*σ*) of each fitted function. The functions are stacked in descending age order (age listed in center). The black dotted line represents the exponential regression fit to the BIC DN1 vs. ITD curve widths, showing a decrease in width with age. (g**–**k) Development of BIC DN1 amplitude vs. ITD fitted functions. (g) BIC DN1 amplitude vs. ITD data were fit with a 4-parameter Gaussian (*σ*, the curve width; C, the curve center; A, the modulation of the curve; B, the baseline value). Spearman’s rho revealed a significant age-related decrease in curve width (**h**; correlation = −0.63, *p* = 0.000) but not for the other three parameters (**i–k**; correlations = −0.34 to −0.02, *p* = 0.032–0.901). An exponential decay function was fit to the curve width data (h; red line). (l) The BIC DN1 peak derives from an excitatory (E)–inhibitory (I) computation present in the LSO [48]. BIC DN1 peak amplitude is maximum at 0 µs ITD, which corresponds to the greatest E–I overlap (i.e., largest reduction in the binaural waveform). The grey shaded area corresponds to the acoustical ITD range for the guinea pig (250−320 µs) [60]. (m**–**n) A model of binaural interaction in LSO cells was used with two parameters: sigma (*σ*), representing the precision of excitatory and inhibitory input timing, and the inhibition window (*w*), representing the duration of the inhibition. The inhibition window (*w*) decreased significantly with age (**m**; correlation = −0.49; *p* = 0.002), while *σ* did not (**n**; *p* = 0.835). An exponential decay function (red line) was fit when a significant correlation was observed. All fits had *R*^2^ values > 0.7, with the majority >0.9. The data underlying this figure can be found at https://doi.org/10.17605/OSF.IO/KAW34.

We next characterized binaural function using the ABR measurements. Since the ABR includes evoked responses from the superior olivary complex (Fig 2A), where the initial processing of the binaural acoustical cues occurs [32], it is possible to derive a binaural-specific component that is separate from the monaural response. Due to the monaural origin of the early ABR waves I–III, the magnitude of the peaks observed in response to binaural clicks (i.e., presented to both ears) is equal to the summed magnitudes in response to monaural clicks (Fig 2C). Later peaks that are binaural in origin (e.g., wave IV) are not equal to the sum of their monaural ABRs, indicating a binaural-specific computation [61]. This difference is referred to as the binaural interaction component (BIC; Fig 2C), which originates from the LSO in the superior olivary complex [48,50]. Here, we use the DN1 peak of the BIC for our analyses, which is altered in patients with binaural hearing dysfunction despite having normal audiological hearing [47].

The derived binaural-specific BIC of the ABR displayed a significant decrease in latency with age (*r*_*s*_ = −0.85, *n* = 39, *p* = 0.000), similar to the developmental time course of the later ABR waves (Fig 2D). No significant change in BIC amplitude with age was observed (*p* = 0.054; Fig 2E). Given that the source of the BIC, the LSO, comprises a distinct excitatory–inhibitory (EI) interaction from each ear input, it is possible to use the BIC to assess EI function by varying the timing of clicks presented to each ear (i.e., interaural time difference) [49,51]. When there is no temporal disparity between left and right ear clicks (0 μs ITD), the BIC amplitude is maximal and decreases with non-zero ITDs [62] (Fig 2F). Here, we presented clicks with a range of ITDs (±2,000 μs) to characterize BIC amplitude as a function of ITD. The data are then fit with a 4-parameter Gaussian function to quantify: [1] the ITD of maximal BIC, [2] the curve width (*σ*) of the Gaussian function, [3] the modulation of the curve (i.e., amplitude), and [4] the baseline BIC value (Fig 2G). Spearman’s rho correlation analysis revealed a significant decrease in the curve width with age (*r*_*s*_ = −0.63, *p* = 0.000), but not for the other three parameters (correlations: −0.34 to −0.02, *p* = 0.032–0.901). Fig 2F shows individual BIC versus ITD functions for each time point collected, along with horizontal lines showing the decreasing curve width (*σ*) with age (black dotted line). Newborn animals (P1) exhibit broad BIC versus ITD curves. As animals age, curve widths narrow and stabilize after approximately P40.

To determine the source of the narrowing of BIC versus ITD curve width with age, we used a previously established model of the BIC [48] to pinpoint whether: [1] the arrival time and resultant interaction of excitatory and inhibitory inputs to the LSO becomes more precise with arrival times of EI inputs, and/or [2] the window of EI integration shortens as the auditory brainstem matures (Fig 2I–2N). The model fit the data well (*R*^2^ 0.7–0.99) and revealed the EI integration window in LSO cells significantly decreased with age from approximately 0.5 ms in newborn animals to approximately 0.3 ms at ages >P40 (Fig 2M; *r*_*s*_ = −0.49, *p* = 0.002). Age had no significant effect on the precision of EI arrival time (*r*_*s*_ = −0.04, *p* = 0.835; Fig 2N).

In summary, ABR assessments across age revealed the maturation of the guinea pig auditory brainstem follows a similar developmental time-course as the acoustical cues to sound location [59] where values are stable by P56. Therefore, this provides the precise duration needed to induce a unilateral hearing loss that spans the developmental period of the binaural auditory brainstem (i.e., BIC).

### Early unilateral hearing loss alters the binaural interaction component of the ABR

**Fig 3:**
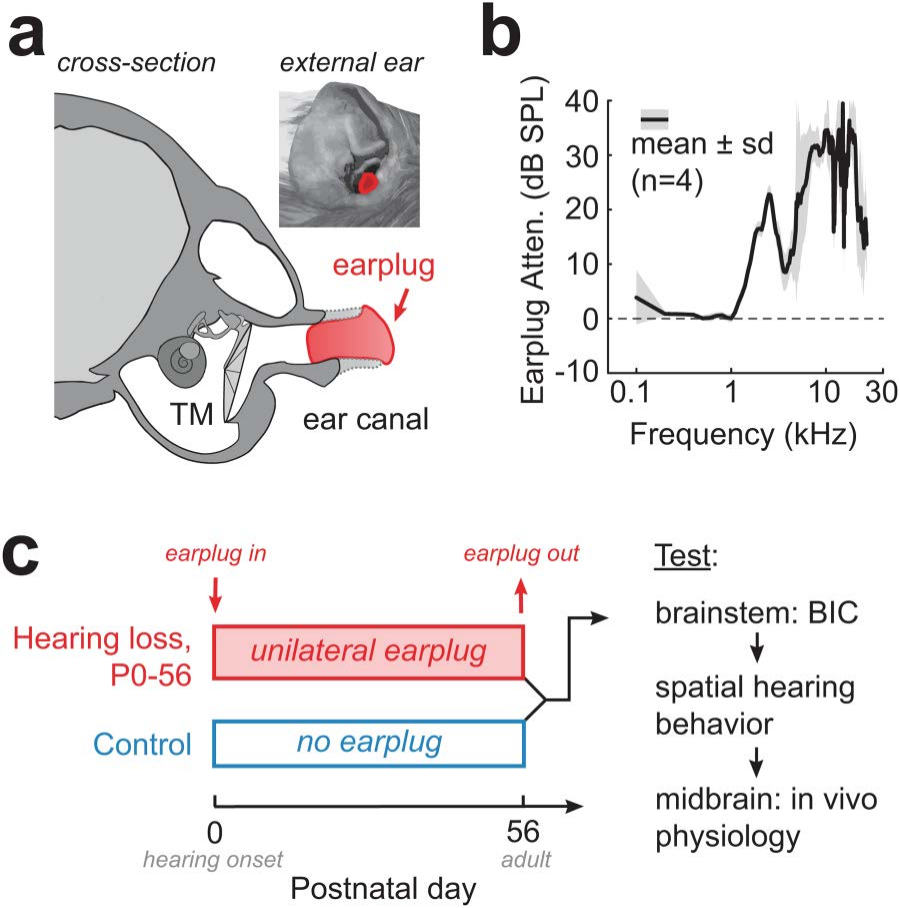
Transient conductive hearing loss (CHL) during development. (a) Schematic of coronal section of a guinea pig skull (traced from high-resolution CT scan). A custom-made earplug is fitted within the cartilaginous ear canal (dotted line), a safe distance from the tympanic membrane (TM). *Inset*: View of external ear with earplug in place. (b) Earplugs provide 10–35 dB SPL sound attenuation for frequencies >1 kHz. (c) Experimental timeline. Litters of newborn guinea pigs were divided into two groups at hearing onset (birth): pups that were raised with no earplug (littermate Controls) and pups that were raised with a unilateral earplug (“Early CHL”). Earplugs remained in place until adulthood (P56). Following earplug removal, animals were tested on: auditory brainstem responses (ABRs) including the binaural interaction component (BIC), a startle-based spatial discrimination task, and in vivo single-unit recordings in the auditory midbrain (i.e., inferior colliculus, IC). All testing was done within 1 week of earplug removal.

Since the auditory brainstem of the guinea pig matures over a prolonged period, this suggests that the binaural circuits may be vulnerable to sensory deprivation during this time. Thus, we hypothesized that a hearing loss in one ear would disrupt the normal development of binaural circuits that depend on input from both ears. To test this, we induced a reversible, mild-moderate hearing loss in one ear using custom earplugs (Fig 3A and 3B) from P0 (birth) through adulthood (P56) in guinea pigs. When animals reached P56, the earplug was removed, and normal auditory input was restored. The earplug manipulation was used as it allows for a conductive hearing loss (CHL) that is reversible and without permanent disruption of cochlear function [63]. Animals that received a developmental CHL (*n* = 21) were designated as “Early CHL” and compared to littermate controls that did not receive an earplug (“Control”; *n* = 38). Following earplug removal, animals underwent a battery of assessments within 1 week of earplug removal: auditory brainstem responses (ABR, BIC), startle-based spatial discrimination, and in vivo single-unit recordings in the auditory midbrain (see timeline in Fig 3C).

ABRs were obtained in Early CHL and Control animals after earplug removal. First, we verified whether normal audibility was restored following earplug removal. We examined click-evoked monaural ABRs and determined ABR thresholds for each animal (see example in S1A Fig). For Early CHL animals, monaural ABRs from the ear ipsilateral to the previous CHL are designated as “Early CHL_ipsi_” and “Early CHL_contra_” for monaural ABRs from the ear contralateral to the previous CHL. Indeed, both normal hearing and CHL-reared animals displayed comparable click-evoked monaural ABR thresholds (median ± SD: Control: 35 ± 7.2 dB SPL; Early CHL_ipsi_: 40 ± 7.9 dB SPL; Early CHL_contra_: 35 ± 5.8 dB SPL; Mann–Whitney U: *p* > 0.01; S1B Fig). Developmental CHL did not significantly alter ABR wave I latencies compared to Controls (Mann–Whitney U: *p* > 0.05; S1C Fig). The latencies for ABR waves III and IV were comparable between Control and Early CHL_contra_ (Mann–Whitney U: *p* > 0.05; S1D and S1E Fig), though Early CHL_ipsi_ displayed longer latencies for monaural ABR waves III and IV (Mann–Whitney U; wave III: *p* < 0.05; wave IV: *p* < 0.01; S1D and S1E Fig). Developmental CHL also did not significantly alter monaural ABR wave I–IV amplitudes compared to Controls (Mann–Whitney U: *p* > 0.05; S1F–S1H Fig).

Since hearing loss has been reported to alter the latency of auditory brainstem responses of binaural nuclei [45,64–66], we first opted to examine BIC latency in Control and Early CHL animals. We find the median (±SD) latency for Controls to be 3.25 ± 0.19 ms, and for Early CHL to be 3.38 ± 0.18 ms (S2 Fig). A Mann–Whitney U test revealed these latencies to be significantly different from one another (*z* = 2.13, *p* = 0.033), with Early CHL animals displaying, on average, 0.13 ms longer BIC latencies than Controls.

**Fig 4:**
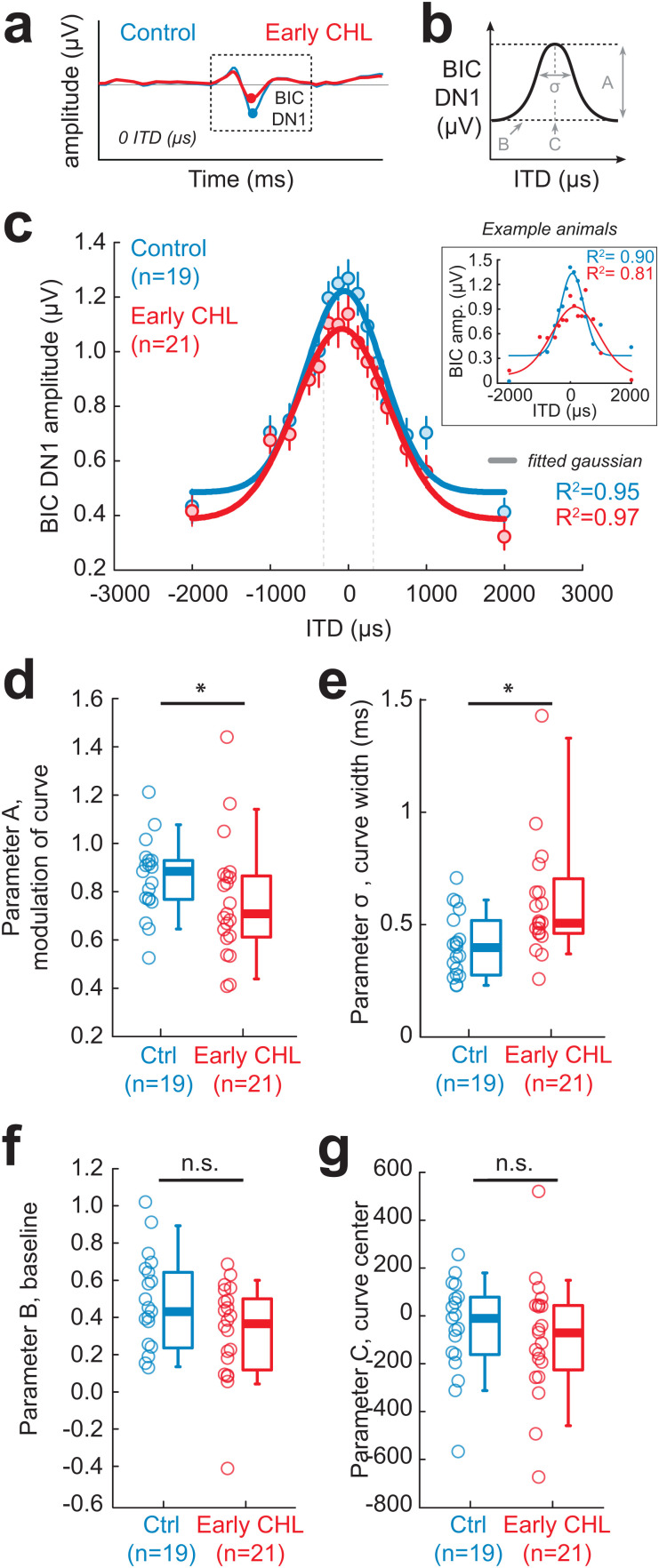
Unilateral developmental CHL alters the binaural interaction component of the ABR. (a) Example Control (blue) and Early CHL (red) binaural interaction component (BIC) at 0 ITD (µs). Circles indicate the BIC DN1 peak. (b) BIC DN1 amplitude vs. ITD data are fit with a 4-parameter Gaussian model. The parameters include: Parameter A, the modulation of the curve; Parameter B, baseline; Parameter C, the curve center; Parameter *σ*, curve width. (c) The average BIC DN1 amplitudes (µV; circles ± SEM) were computed at each ITD tested (±2 ms) for Control (*n* = 19) and Early CHL animals (*n* = 21). Gaussian functions were fit to the average BIC amplitude vs. ITD for each group (thick lines). The dotted grey vertical lines indicate the acoustic ITD range for adult guinea pigs (250–320 µs) [60]. *Inset*: Individual sessions for Control and Early CHL example animals. Dots indicate normalized BIC DN1 amplitude vs. ITD datapoints with individually fitted Gaussian function (thin lines). (d) Early CHL animals display significantly shallower modulation of the fitted Gaussian curves (Parameter A) compared to Controls (Mann–Whitney U, *p* < 0.05). (e) Early CHL animals display significantly broader Gaussian curve widths (*σ*) compared to Controls (Mann–Whitney U, *p* < 0.05). (f, g) There were no significant differences between Early CHL and Controls for Parameter B (baseline) or Parameter C (curve center). Open circles indicate individual data, and boxplots indicate group medians. The data underlying this figure can be found at https://doi.org/10.17605/OSF.IO/KAW34.

Next, we examined BIC tuning to interaural timing disparities (i.e., ITD) to probe underlying excitatory/inhibitory function of the auditory brainstem (Fig 4A and 4B). Fig 4C shows BIC amplitude versus the timing difference click stimuli presented to both ears (±0, 125, 250, 375, 500, 750, 1,000, and 2,000 μs) for Control (*n* = 19 animals) and Early CHL animals (*n* = 21). For both groups, the amplitude is maximal for an ITD of zero (i.e., no timing difference between ears) and decreases with larger ITDs (i.e., larger timing differences between ears). The BIC versus ITD data is fit with a 4-parameter Gaussian function to quantify key aspects of the BIC amplitude and ITD relationship (Fig 4B): the ITD of maximal BIC, the curve width of the Gaussian function, the modulation of the curve (e.g., amplitude), and the baseline BIC value. The average BIC amplitude (±SEM) was computed at each ITD for Controls and Early CHL measurements and a Gaussian function was fit to each group average (Fig 4C). For each animal, the BIC (µV) versus ITD (µs) was fit with the Gaussian (see Fig 4C inset for individual examples) to extract the 4-parameters. Of the four parameters, there is a significant difference between Controls and Early CHL for parameters A (modulation of the curve, *z* = −2.00, *p* = 0.046; Fig 4D) and *σ* (curve width, *z* = 2.95, *p* = 0.0032; Fig 4E), but not for parameters B (baseline, *z* = −1.84, *p* = 0.066; Fig 4F) or C (curve center, *z* = −0.79, *p* = 0.43; Fig 4G). In summary, ABR measurements from Early CHL animals had significantly reduced BIC amplitudes at small ITDs (<250 μs) and significantly broader BIC versus ITD tuning compared to their littermate Controls. These results suggest a unilateral CHL in development alters the maturation of binaural information processing at the level of the auditory brainstem.

### Adult-onset CHL does not disrupt the binaural interaction component of the ABR

**Fig 5:**
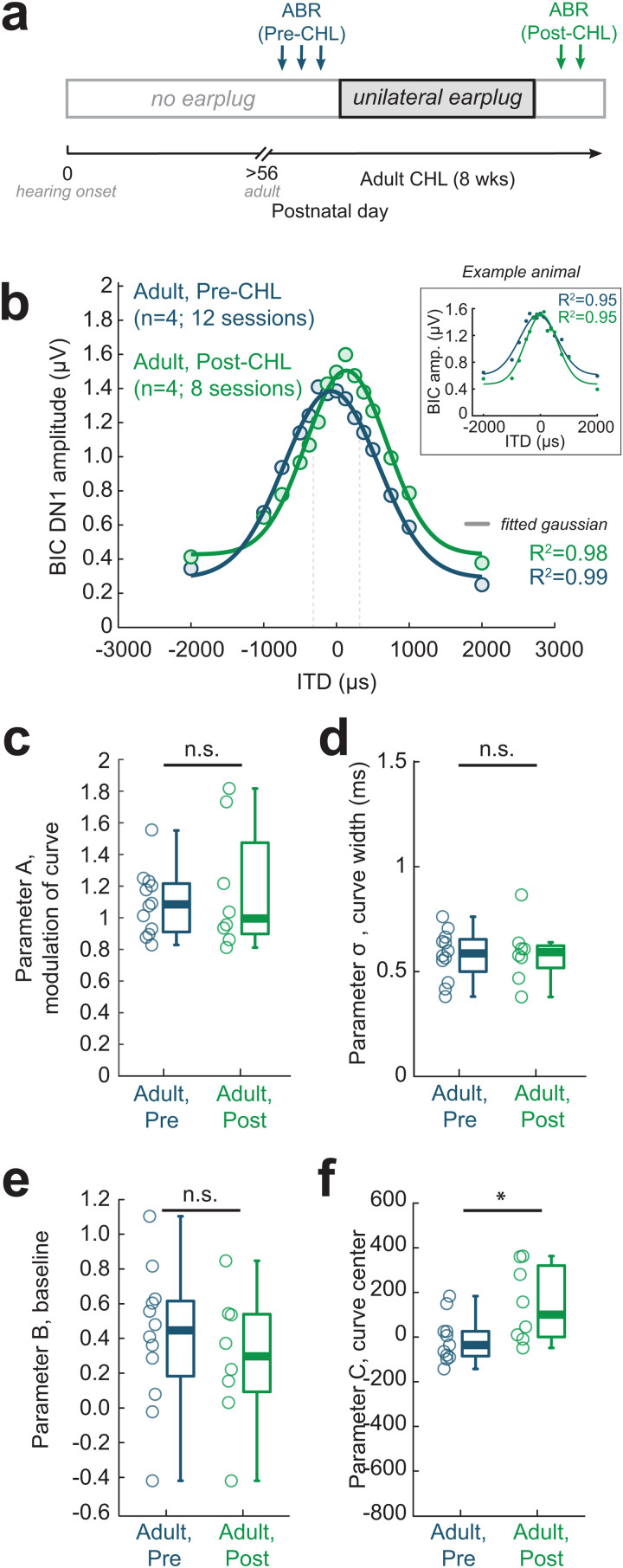
Adult-onset CHL has minimal impact on the binaural interaction component of the ABR. (a) Experimental timeline. ABRs were collected from normal-hearing Adult guinea pigs (>P56) prior to earplug placement (“Pre-CHL”; three sessions per animal). Unilateral earplugs were then placed in each animal for the same duration used in the developmental manipulation (8 weeks). Following earplug removal, ABRs were collected (“Post-CHL”; two sessions per animal). (b) BIC DN1 amplitude (µV) vs. ITD (µs) for Pre-CHL and post-CHL ABR measures. The average for each condition (circles) are fit with a 4-parameter Gaussian model (thick lines). The dotted grey vertical lines indicate the acoustic ITD range for adult guinea pigs (250–320 µs) [60]. *Inset*: Pre-CHL and Post-CHL session examples from one animal. Dots indicate normalized BIC DN1 amplitude vs. ITD datapoints individually fitted Gaussian functions (thin lines). (c**–**f) Adult-onset CHL did not alter Parameter A (modulation of the curve; *p* = 0.97), curve width (*p* = 0.97), or Parameter B (baseline; *p* = 0.46), but did shift the BIC vs. ITD curve center (*p* = 0.04). Open circles indicate individual data, and boxplots indicate group medians. The data underlying this figure can be found at https://doi.org/10.17605/OSF.IO/KAW34.

Although we observed BIC alterations following 8 weeks of Early CHL, it is possible that the duration of CHL alone, at any age range, could lead to the same outcome. To test this, we induced unilateral CHL of the same duration as the Early CHL group (8 weeks) in adult guinea pigs (>P56; *n* = 4) and measured BIC versus ITD tuning. Adults served as within-animal controls, where ABRs were collected prior to earplug placement (“Pre-CHL”; 3 sessions per animal) to establish baseline. Earplugs were then placed for 8 weeks, removed, and post-CHL ABR assessments were made (“Post-CHL”; 2 sessions per animal; see timeline in Fig 5A). Fig 5B shows the average BIC amplitude (µV) as a function of ITD (µs) for Pre- and Post-CHL assessments (circles), along with the fitted 4-parameter Gaussian (thick lines). For each session, the BIC amplitude (µV) versus ITD (µs) was fit with the Gaussian (see Fig 5B inset for individual examples) to extract the 4-parameters for each Pre- and Post-CHL condition. We find that Adult-onset CHL did not significantly alter Parameter A (modulation of the curve; *p* = 0.97; Fig 5C), curve width (*p* = 0.97; Fig 5D), or Parameter B (baseline; *p* = 0.46; Fig 5E) but did shift the curve center of the BIC versus ITD function (*p* = 0.04; Fig 5F). This contrasts with the significant shifts observed following Early CHL with Parameter A (modulation of curve) and the curve width. Thus, we interpret the BIC alterations observed following Early CHL as potentially reflecting a developmental vulnerability to CHL.

### Early unilateral hearing loss disrupts spatial discrimination of high- but not low-frequency sounds

Since the binaural component of the ABR (i.e., BIC) was altered by Early CHL, we asked whether Early CHL animals also displayed impairments in auditory behavior that requires binaural hearing. Previously employed behavioral assays are not optimal for our developmental study, as they use operant conditioning or other methods that require training that can take months [57,67,68] and are difficult to employ in some rodents including guinea pigs [69]. Therefore, we opted for an alternative approach that requires no training: a startle reflex-based method that can be used to assess spatial hearing ability in guinea pigs [70].

**Fig 6:**
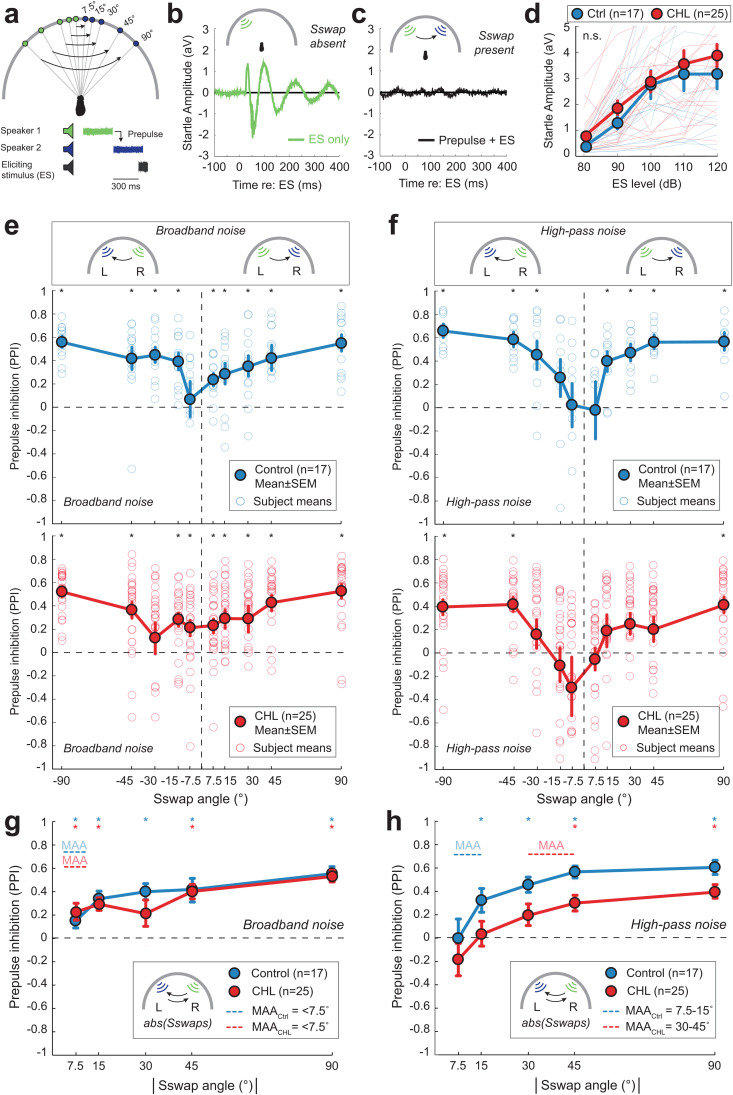
Unilateral developmental CHL alters spatial discrimination of high-pass noise. (a) Schematic of the startle-based behavioral paradigm to assess spatial discrimination. A continuous noise stimulus (broadband or high-pass noise) is presented from one speaker (Speaker 1, green) then switches to a second speaker (Speaker 2, blue) for a short interstimulus interval (ISI, 300 ms) prior to presentation of a brief startle-eliciting stimulus (ES; 110−120 dB SPL, 20 ms). The direction of the speaker swap (“Sswap”) is randomized (i.e., left-to-right, right-to-left). The angle of the speaker swap prepulse varied from 7.5° to 90° (7.5°, 15°, 30°, 45°, 90°). Note, for CHL animals, the left side is ipsilateral to the previous earplug. (b) Example startle response for the control condition where no prepulse was presented (ES only, no speaker swap). (c) Example response when a prepulse is presented prior to ES (Prepulse + ES). In this example, the animal detected the change in speaker locations resulting in a reduced startle response (Prepulse inhibition, PPI). (d) Startle response amplitude (arbitrary volts, aV) as a function of ES level (dB SPL) in the presence of background noise (70 dB SPL) for Control (*n* = 17) and Early CHL (*n* = 25) animals. There are no significant differences between groups in baseline startle responses (*p* > 0.05). (e) Prepulse inhibition (PPI) as a function of speaker swap angle of broadband noise (100 Hz–20 kHz) for Controls (top panel, blue) and CHL animals (bottom panel, red). Data are shown for both right-to-left and left-to-right speaker swap conditions. PPI greater than 0 indicates detection of the speaker swap prepulse. Open circles are the individual subject means, and filled circles are the group mean ± standard error of the mean (SEM). Asterisks indicate the angle at which the population mean response is significantly different from 0 (*= *p* < 0.05). (f) PPI as a function of speaker swap angle of high-pass noise (<2 kHz) for Controls (top panel, blue) and Early CHL animals (bottom panel, red). (g) Mean PPI (±SEM) for Control (*n* = 17) and Early CHL (*n* = 25) using the broadband noise prepulse for left-to-right and right-to-left speaker swaps combined. The minimum audible angle (MAA) is defined as the smallest angle at which the group-level PPI is significantly different from 0. Horizontal dotted lines show the MAA range for Control and Early CHL animals. For broadband noise, Control and Early CHL animals can both discriminate speaker swaps of ≥7.5°. (h) Mean PPI (±SEM) for Control (*n* = 17) and Early CHL (*n* = 25) using the high-pass noise prepulse for left-to-right and right-to-left speaker swaps combined. For high-pass noise, Control animals can discriminate speaker swaps between 7.5° and 15°, whereas Early CHL animals discriminate between 30° and 45°. The data underlying this figure can be found at https://doi.org/10.17605/OSF.IO/KAW34.

Behavioral spatial acuity was assessed using a speaker-swap paradigm [70,71] (Fig 6A), which allowed us to determine the smallest angle for which a change in speaker location could be detected by the animal. Broadband or high-pass noise was used to probe stimulus detection using acoustical cues available to the guinea pig. In the horizontal plane, broadband noise provides access to both interaural time and level difference cues (ITD, ILD), whereas high-pass noise largely provides access to ILD cues only. ILD cues are initially encoded by the LSO [72], the source of the BIC of the ABR. Guinea pigs reflexively startle in response to loud, unexpected sounds (Fig 6B), but presentation of a detectable cue (a “prepulse”) prior to the loud sound leads to a reduced startle response (Fig 6C). This is known as prepulse inhibition (PPI) of the acoustic startle response [73]. Here, the prepulse is a shift of continuous noise stimulus (broadband or high-pass) presented from one speaker to a second speaker for a short interstimulus interval (ISI, 300 ms) prior to presentation of a startle-eliciting stimulus (via speaker mounted above animal). The direction of the swap was randomized (e.g., noise swaps from left-to-right or right-to-left speakers).

Prior to testing with the speaker swap paradigm, baseline startle responses were characterized for all animals to determine whether Control and Early CHL animals displayed comparable startle responses. Fig 6D shows the mean startle response amplitude (±SEM) as a function of increasing sound intensity (dB SPL) of the startle speaker in the presence of 70 dB SPL background noise for Control (*n* = 17) and Early CHL animals (*n* = 25). A two-way ANOVA was performed to examine the effect of group (Control versus CHL) and sound level on startle response amplitude. There was not an effect of group on response amplitudes (*F*_1,210_ = 2.9, *p* = 0.09), indicating no significant differences between Control and Early CHL groups across all sound levels. However, there was a significant effect of sound level (*F*_4,210_ = 20.6, *p* < 0.0001). The interaction between group and sound level was not significant (*F*_4,210_ = 0.15, *p* = 0.96). Tukey’s HSD post hoc test revealed significant differences in startle response amplitudes between 80 dB SPL and all higher levels (90, 100, 110, 120 dB SPL; all: *p* < 0.05) and between 90 dB SPL and all higher levels (100, 110, 120 dB SPL; all: *p* < 0.05). Thus, both groups exhibited comparable baseline startle responses prior to testing on the speaker swap paradigm (testing at 110–120 dB SPL).

Spatial acuity in the frontal field was assessed by swapping the location of a continuous broadband or high-pass noise symmetrically across the midline between speakers separated by ±90°, 45°, 30°, 15°, and 7.5° (from the left-to-right and from the right-to-left) preceding the startle-eliciting stimulus (ES; 110–120 dB SPL; 300 ms ISI). Control conditions, in which no swap occurred, were presented from each of the five starting speaker locations (i.e., noise presented from speaker 1, but did not swap to a second speaker prior to ES). Fig 6E and 6F shows the results of varying speaker swap angle across midline, where the PPI (group mean ± SEM) for each angle condition is shown as a function of angle for broadband or high-pass noise speaker swaps for Control animals (top panels) and Early CHL animals (bottom panels). Open circles indicate the mean PPI for each subject, at each angle tested. A PPI value of zero indicates that the startle response for the control condition (e.g., no speaker swap) is equal to the startle response with the prepulse present (e.g., speaker swap present), indicating that the swap in angle was not detected. Thus, PPI values that are significantly different than zero indicate that the animal was able to detect the speaker swap. To assess significance, a repeated-measures ANOVA was used to determine whether PPI was significantly different from zero across all angles, followed by Bonferroni-corrected two-tailed Student’s *t*-tests to identify specific angles where PPI was significantly different from 0. The smallest angle that is significantly different from zero is defined as the minimum audible angle (MAA) (cf. [74]).

The broadband noise condition (Fig 6E) affords access to both low-frequency ITD and high-frequency ILD cues. For Controls (*n* = 17; Fig 6E, *top*) a repeated-measures ANOVA revealed significant effect of angle on PPI (*F*_9,108_ = 4.4, *p* < 0.0001). Two-tailed Student’s post hoc *t t*est with Bonferroni correction revealed Control group PPI was significantly different from PPI = 0 (i.e., compared to control no prepulse conditions) for left-to-right speaker swaps at all angles (7.5°, 15°, 30°, 45°, and 90°; *t* = 3.3 − 7.8; *p* = 0.0001 − 0.02) and for righ*t*-to-left speaker swaps at −15°, −30°, −45°, and −90° (*t* = 4.6 − 13.9; *p* = 9.2 × 10^−8^ − 0.006). For Early CHL animals (*n* = 25; Fig 6E, *bottom*), a repeated-measures ANOVA also revealed a significant effect of angle on PPI (*F*_9,216_ = 3.8, *p* = 0.0002). Post hoc analysis (two-tailed Student’s *t t*est) with Bonferroni correction revealed Early CHL group PPI was significantly different from PPI = 0 for left-to-right speaker swaps at all angles (7.5°, 15°, 30°, 45°, and 90°; *t* = 2.7 − 9.3; *p* = 2.2 × 10^−8^ − 0.05) and for righ*t*-to-left speaker swaps at −7.5°, −15°, −45°, and −90° (*t* = 3.3 − 14.6; *p* = 1.9 × 10^−9^ − 0.03).

To determine the MAA for broadband stimuli, we opted to collapse the left-to-right and right-to-left speaker swaps (i.e., absolute value of speaker angle) for Control and Early CHL animals (Fig 6G). For Controls, a repeated-measures ANOVA revealed significant effect of angle on PPI (*F*_4,48_ = 7.7, *p* < 0.0001), followed by two-tailed Student’s post hoc *t* test with Bonferroni correction revealed PPI was significantly different from zero at all broadband noise speaker swap angles (7.5°, 15°, 30°, 45°, 90°; *t* = 3.4 − 11.2; *p* = 5.3 × 10^−7^ − 0.03). Given that all angles tested were significantly different from PPI = 0 for broadband noise, the group-level MAA for Controls is less than 7.5°. For Early CHL, a repeated-measures ANOVA revealed significant effect of angle on PPI (*F*_4,96_ = 7.4, *p* < 0.0001), followed by post hoc analysis (two-tailed Student *t* test) Bonferroni correction revealed PPI was significantly different from zero for broadband noise speaker swap angles of 7.5°, 15°, 45°, and 90° (*t* = 5.3 − 12.8; *p* = 1.7 × 10^−8^ − 0.0001). Given tha*t* nearly all angles, including the smallest ones tested (7.5° and 15°), were significantly different from PPI = 0, the group-level MAA for Early CHL animals is less than 7.5° for broadband noise. This result suggests that Control and Early CHL animals exhibit similarly small MAAs for broadband noise.

The high-pass noise condition (Fig 6F) affords access to primarily ILD cues. For Controls (*n* = 17; Fig 6F, *top*) a repeated-measures ANOVA revealed significant effect of angle on PPI (*F*_9,81_ = 4.0, *p* = 0.0003). Two-tailed student’s post hoc *t* test with Bonferroni correction revealed Control group PPI was significantly different from PPI = 0 for left-to-right speaker swaps at all angles greater than or equal to 15° (*t* = 4.8 − 11.2; *p* = 9.9 × 10^−5^ − 0.01) and for right-to-left speaker swaps at all angles greater than or equal to −30° (*t* = 3.8 − 14.7; *p* = 1.3 × 10^−6^ − 0.04). For Early CHL animals (*n* = 25; Fig 6F, *bottom*), a repeated-measures ANOVA also revealed a significant effect of angle on PPI (*F*_9,224_ = 7.6, *p* < 0.0001). Post hoc analysis (two-tailed Student’s *t t*est) with Bonferroni correction revealed Early CHL group PPI was significantly different from PPI = 0 for left-to-right speaker swaps at 90° only (*t* = 6.4; *p* = 9.5 × 10^−6^) and for righ*t*-to-left speaker swaps at −45°, and −90° only (*t* = 6.6 − 8.6; *p* = 6.2 × 10^−6^ − 7.1 × 10^−6^).

To determine the MAA for high-pass noise stimuli, we opted to collapse the left-to-right and right-to-left speaker swaps (i.e., absolute value of speaker angle) for Control and Early CHL animals (Fig 6H). For Controls, a repeated-measures ANOVA revealed significant effect of angle on PPI (*F*_4,36_ = 9.2, *p* < 0.0001), followed by two-tailed student’s post hoc *t* test with Bonferroni correction revealed PPI was significantly different from zero at high-pass noise speaker swap angles of 15°, 30°, 45°, and 90° (*t* = 3.4 − 15.8; *p* = 1.9 × 10^−6^ − 0.03). Given *t*hat angles greater than 7.5° were significantly different from PPI = 0 for high-pass noise, the group-level MAA for Controls is between 7.5° and 15°. For Early CHL, a repeated-measures ANOVA revealed significant effect of angle on PPI (*F*_4,100_ = 18.3, *p* < 0.0001), followed by post hoc analysis (two-tailed Student’s *t* test) Bonferroni correction revealed PPI was significantly different from zero for high-pass noise speaker swap angles of 45°, and 90° (*t* = 5.5 − 8.9; *p* = 5.8 × 10^−6^ ). Given tha*t* angles greater than 30° were significantly different from PPI = 0, the group-level MAA for Early CHL is between 30° and 45° for high-pass noise. Thus, on average, the Early CHL group required larger angles of separation to elicit significant PPI to high-pass noise, with group-level MAAs falling between 30° and 45°, compared to 7.5° − 15° for Controls.

### Early unilateral hearing loss alters neural coding of interaural level difference (ILD) cues in the auditory midbrain

Since interaural level difference (ILD) cues to sound location largely depend on high-frequencies, and Early CHL animals display poorer spatial acuity for sounds comprised of high-frequencies, we next asked whether the spatial deficits could be attributed to impaired ILD processing in auditory neurons. Indeed, Early CHL animals displayed alterations in the binaural auditory brainstem response (ABR), of which the derived binaural component is generated by the lateral superior olive (LSO) [48], the site that first integrates binaural information and encodes ILD cues [33]. Further, the PPI of the acoustic startle is mediated by the auditory midbrain (i.e., inferior colliculus) [73], which receives direct inputs from the LSO and contains neurons that encode ILD [72]. Therefore, we directly assessed ILD coding in the central nucleus of the inferior colliculus (IC), from the same animals that displayed BIC (Fig 4) and behavioral spatial hearing deficits (Fig 6). We collected single-unit extracellular recordings from IC neurons from littermate Control and Early CHL animals. For Early CHL, recordings were made in both the IC contralateral (CHL_contra_) and ipsilateral (CHL_ipsi_) to the previously occluded ear. For each isolated IC neuron, the characteristic frequency (CF), rate-level function at CF, and binaural sensitivity to ILD was assessed. Neurons were selected for further analysis if they met a set of criteria for ILD sensitivity (see “Methods”).

**Fig 7:**
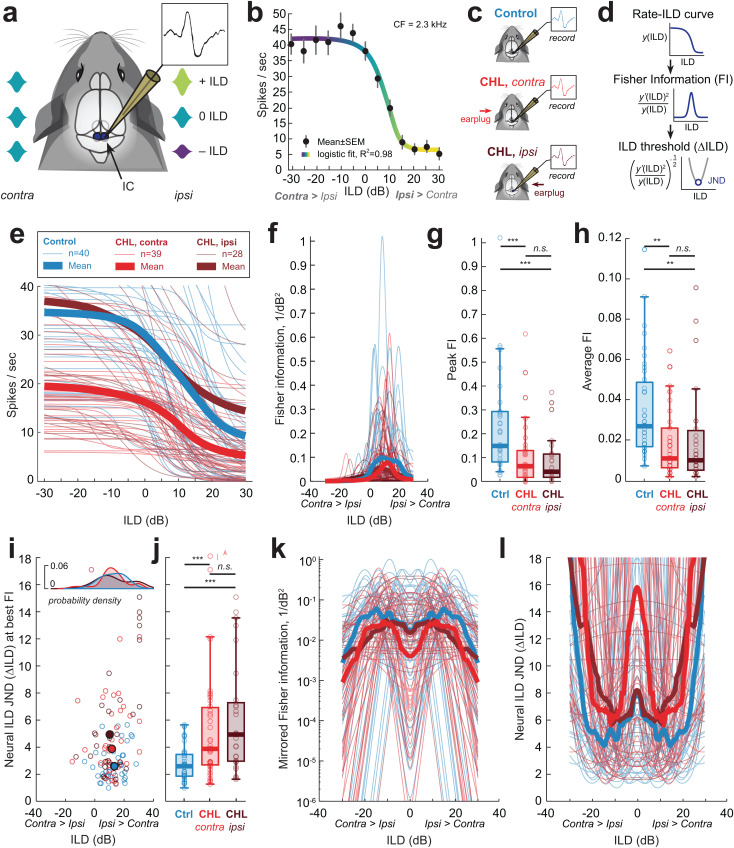
Unilateral developmental CHL degrades neural discrimination of interaural level difference (ILD) cues in auditory midbrain neurons. (a) Schematic of experimental approach. Single-unit extracellular recordings were collected from inferior colliculus (IC) neurons and assessed for binaural sensitivity to interaural level difference (ILD) cues. The sound level of a tone (at the neuron’s characteristic frequency, CF, 20 dB above threshold) is fixed at the ear contralateral to the recording site, and the sound level for the ipsilateral ear (i.e., providing the inhibitory drive) is varied (±30, 5 dB steps). (b) Rate-ILD function for an example ILD-responsive IC neuron. Neurons are classified as ILD-sensitive when firing rates are modulated by ≥50% with increasing sound level to the ipsilateral ear. (c) Overview of recording conditions for Control and Early CHL animals. Neurons recorded from the IC contralateral to the previous CHL are designated as “CHL, *contra*”. Neurons recorded from the IC ipsilateral to the previous CHL are designated as “CHL, *ipsi*”. (d) Rate-ILD curves from each neuron are used to compute Fisher information (FI), from which neural ILD thresholds (ΔILD) can be computed. The just-noticeable-difference (JND) for ILD is defined as the minimum of the ΔILD function. (e) Rate-ILD functions for single IC neurons from Control (blue; *n* = 40), CHL contra (red; *n* = 39), and CHL *ipsi* (dark red; *n* = 28). Thick lines indicate group means, and thin lines indicate individual neurons. (f) Distribution of FI (1/dB)^−½^ as a function of ILD for Control, CHL *contra*, and CHL *ipsi* neurons. (g) The maximum FI for each distribution was computed for each unit. Control neurons displayed significantly higher peak FI values (median ± SD: 0.15 ± 0.2 (1/dB^2^)) than CHL *contra* (0.06 ± 0.1 (1/dB^2^)), and CHL *ipsi* neurons (0.04 ± 0.1 (1/dB^2^); *p* < 0.001). (h) The average of the FI distribution for each unit. Control neurons displayed significantly higher average FI (0.03 ± 0.2 (1/dB^2^)) than CHL *contra* (0.01 ± 0.02 (1/dB^2^)), and CHL *ipsi* neurons (0.01 ± 0.02 (1/dB^2^); *p* = 0.002). (i) Distribution of neural ILD JNDs for Control (*n* = 40), CHL *contra* (*n* = 39), and CHL *ipsi* (*n* = 28) single units. *X*-axis location indicates the ILD location at which the best FI was observed for that unit. Open circles indicate individual neurons, and filled circles depict the median ILD location (*x*-axis) and corresponding ILD JND (*y*-axis) for each group (Control: ILD location:13 dB ILD, median JND: 2.59 dB ILD; CHL *contra*: location: 11.3 dB ILD, median JND: 3.9 dB ILD; CHL *ipsi*: location: 9.8 dB ILD, median JND: 4.9 dB ILD). *Top inset*: probability density estimates for distribution of ILD location for each group (Control, CHL *contra*, CHL *ipsi*). *X*-axis of distributions align with the *x*-axis for main plot in (j). Single IC neurons from Early CHL animals exhibit significantly elevated neural ILD JNDs compared to Controls (median ± SD: Control: 2.59 ± 1.3 dB ILD; CHL *contra*: 3.9 ± 4.2 dB ILD; CHL *ipsi*: 4.9 ± 4 dB ILD; ***= *p* < 0.001). (k) “Mirrored” population FI as a function of ILD. Each IC cell and corresponding rate-ILD function is assumed to have a mirrored equivalent in the opposite IC. The “left” and “right” IC cells are summed to compute the population FI that incorporates both hemispheres. (l) The neural ILD JND derived from the mirrored population FI in (k). The median (±SD) JND at midline (0 dB ILD) for Control cells is 7.8 ± 164.4 dB ILD, for CHL *contra* cells is 15.8 ± 396.1 dB ILD, and for CHL *ipsi* cells is 8.2 ± 73.8 dB ILD. For all boxplots, the thick center line indicates the median, and the top and bottom of each box are the 75th and 25th percentiles, respectively. The data underlying this figure can be found at https://doi.org/10.17605/OSF.IO/KAW34.

ILD sensitivity was determined by varying the intensity of the tone stimulus (the CF of the isolated unit) presented to both ears through the insert earpieces (Fig 7A). The sound level at the contralateral ear was held constant (i.e., excitatory ear), and the level to the ipsilateral ear (i.e., inhibitory ear) was then varied ±30 dB (5 dB steps). Fig 7B shows an example rate-ILD function for an ILD-sensitive IC neuron. All raw rate-ILD data were fit with a four-parameter sigmoidal logistic function (S3A and S3B Fig). The parameters include the maximum and minimum firing rate (spikes/second, s/s) of the rate-ILD functions, the 50% inflection point of the sigmoid fit (i.e., half-maximum of fit), the ILD dynamic range (i.e., range of ILD producing 10%–90% of maximal firing), and the slope of the rate-ILD functions. IC neurons that met criteria for ILD sensitivity were collected from littermate Controls (16 subjects; *n* = 40 neurons, median = 2.0 neurons per animal, range: 1–9 neurons) and Early CHL animals (*n* = 14 subjects; CHL_contra_: *n* = 39 neurons, 2.0 neurons per animal, range: 1–18 neurons; CHL_ipsi_: *n* = 28 neurons, 3.0 neurons per animal, range: 2–9 neurons).

The median (±SD) maximum firing rates of the ILD functions were 34.6 ± 20.9 s/s for Controls (interquartile range, IQR: 22.8–43.6), 19.5 ± 9.8 s/s for CHL_contra_ neurons (IQR: 14.9–26.4), and 27.6 ± 30.5 s/s for CHL_ipsi_ neurons (IQR: 18.4–54.9; S3C Fig). A Mann–Whitney U test revealed a significant difference between the maximum firing rate of Controls and CHL_contra_ neurons (*z* = 4.77, *p* < 0.0001) but not CHL_ipsi_ neurons (*z* = 0.66, *p* = 0.51). CHL_contra_ neurons were also significantly different from CHL_ipsi_ neurons (*z* = −2.75, *p* = 0.006). For the minimum firing rates of the ILD functions, the median (±SD) were 4.1 ± 13.6 s/s for Controls (IQR: 0.7–9.7), 2.6 ± 4.73 s/s for CHL_contra_ neurons (IQR: 0.6–6.5), and 4.6 ± 21 s/s for CHL_ipsi_ neurons (IQR: 1.3–16.1; S3D Fig). A Mann–Whitney U test found no significant differences between the groups (Controls versus CHL_contra_: *z* = 1.3, *p* = 0.19; Controls versus CHL_ipsi_: *z* = −0.36, *p* = 0.72; CHL_contra_ versus CHL_ipsi_: *z* = −1.44, *p* = 0.15).

For the 50% inflection point of the sigmoid fit, the median (±SD) were 10.4 ± 7.4 dB for Controls (IQR: 3.7–16.7), 9.2 ± 9.8 dB for CHL_contra_ neurons (IQR: 2.9–14.4), and 6.2 ± 9.0 dB for CHL_ipsi_ neurons (IQR: 0–9.5; S3E Fig). A Mann–Whitney U test found no significant differences between the groups (Controls versus CHL_contra_: *z* = 0.59, *p* = 0.56; Controls versus CHL_ipsi_: *z* = 1.96, *p* = 0.05; CHL_contra_ versus CHL_ipsi_: *z* = 1.61, *p* = 0.11). For the ILD dynamic range of the sigmoid fit, the median (±SD) were 15 ± 11.7 dB for Controls (IQR: 10.4–16.7), 22.2 ± 13.8 dB for CHL_contra_ neurons (IQR: 10.6–29.9), and 23.5 ± 16.5 dB for CHL_ipsi_ neurons (IQR: 19.8–39.9; S3F Fig). A Mann–Whitney U test revealed a significant difference between the ILD dynamic range of Controls and CHL_ipsi_ neurons (*z* = −2.99, *p* = 0.0028) but not CHL_contra_ neurons (*z* = −1.37, *p* = 0.17). There were no significant differences between CHL_contra_ and CHL_ipsi_ neurons (*z* = −1.65, *p* = 0.099). Finally, the slope of the rate-ILD functions were found to be significantly smaller in both CHL groups compared to Controls (S3G Fig). The median slopes (±SD) were 3 ± 3.6 sp/s/dB for Controls, 0.6 ± 1.15 sp/s/dB for CHL_contra_ neurons, and 0.58 ± 3.4 sp/s/dB for CHL_ipsi_ neurons. A Mann–Whitney U revealed significant differences between Controls and CHL groups (Controls versus CHL_contra_: *z* = 5.2, *p* < 0.00001; Controls versus CHL_ipsi_: *z* = 3.28, *p* = 0.001) and no significant differences between CHL_contra_ and CHL_ipsi_ neurons (*z* = −0.43, *p* = 0.67).

In summary, Early CHL animals exhibit the following differences in rate-ILD functions of midbrain neurons: [1] a reduction in maximum firing rate for both CHL_contra_ and CHL_ipsi_, [2] an increase in ILD dynamic range (dB) for CHL_ipsi_, and [3] shallower ILD slopes (sp/s/dB) for both CHL_contra_ and CHL_ipsi_.

### Early unilateral hearing loss degrades neural discrimination of interaural level difference (ILD) cues in the auditory midbrain

Other developmental plasticity studies of the auditory system have also reported changes to rate-ILD coding in the auditory midbrain [22,24,26]. But, it is uncertain how these changes relate to a behavioral outcome. Here, using the startle-based behavioral paradigm, Early CHL animals displayed impairments with discriminating sound sources that rely on ILD cues. We employed the mathematical framework of Fisher Information (FI) to compute neural ILD discrimination values of IC neurons [75–79], enabling us to compare neurophysiological and behavioral discrimination of sound sources.

Fig 7E shows rate-ILD functions for all IC neurons collected from littermate Controls (*n* = 40) and Early CHL animals (contra: *n* = 39; ipsi: *n* = 28) that met the criteria for ILD sensitivity (see “Methods”). The distribution of FI is drawn from the rate-ILD functions (e.g., see Fig 7D). Fig 7F shows the distribution of FI across ILDs (±30 dB ILD) for ILD sensitive IC neurons. The maxima for the FI distribution for each unit was taken and compiled in a summary boxplot (Fig 7G). The median FI for CHL_contra_ neurons is 0.06 ± 0.1 (SD) 1/dB^2^, 0.04 ± 0.1 for CHL_ipsi_ neurons, and 0.15 ± 0.2 for Control neurons. We find both CHL groups exhibit a significant reduction in peak FI values compared to Controls (Mann–Whitney U, Controls versus CHL_contra_: *z* = 3.9, *p* = 0.0001; Controls versus CHL_ipsi_: *z* = 4.04, *p* < 0.00001). Peak FI between the two CHL groups was not significantly different (*z* = 0.74, *p* = 0.46).

Next, we quantified neural ILD sensitivity by computing neural ILD discrimination thresholds (i.e., the smallest change in ILD that a neuron can detect, given a specific criterion). FI can be directly related to the well-known discriminability index, *d*′, a signal detection metric used to quantify discrimination of sensory stimuli [75,77–81]. Here, we use FI to evaluate the change in ILD necessary to achieve 1 standard separation in spiking distributions (i.e., the neural just-noticeable difference, JND, for a *d*′* *= 1). In this formula, higher FI values correspond to lower neural ILD thresholds (i.e., better ILD discrimination). Fig 7I shows the ILD JNDs for Control and Early CHL neurons at the ILD location of its best FI (location of peaks in Fig 7F). The median neural ∆ILD for Control neurons is 2.6 ± 1.3 dB ILD (median ± SD), for CHL_contra_ neurons is 3.9 ± 4.2 dB ILD and for CHL_ipsi_ neurons is 4.9 ± 4 (Fig 7J). We find both Early CHL groups exhibit significantly elevated ILD JNDs (approximately 1−2 dB ILD worse) as compared to Controls (Controls versus CHL_contra_: *z* = −3.9, *p* = 0.0001; Controls versus CHL_ipsi_: *z* = −4.04, *p* < 0.00001). Early CHL groups were not significantly different from each other (*z* = −0.74, *p* = 0.46).

Assuming ILD discrimination depends on neural populations from both left and right IC nuclei, we next opted to pool the data to reflect FI and JND that incorporates both hemispheres (Fig 7K and 7L). Each IC cell and corresponding rate-ILD function is assumed to have a mirrored equivalent in the opposite IC. The “left” and “right” cells are summed to compute the population FI that includes both hemispheres (“Mirrored FI”; Fig 7K). Both CHL_contra_ and CHL_ipsi_ neurons display lower FI at non-zero ILDs compared to Control neurons. The median FI across all ILDs (±30 dB ILD) were lower in CHL neurons (Control: 0.03; CHL_contra_: 0.011; CHL_ipsi_: 0.012). At midline (0 dB ILD), CHL_contra_ neurons have lower FI than CHL_ipsi_ and Controls (Control: 0.018; CHL_contra_: 0.004; CHL_ipsi_: 0.015). The neural ILD JND is then recomputed based on the Mirrored FI to generate the population JND that represents neurons from both IC nuclei (Fig 7L). Both CHL neuron types displayed poorer ILD JNDs for non-zero ILDs. The median JND across all ILDs (±30 dB ILD) were elevated in CHL neurons (Control: 5.9; CHL_contra_: 9.3; CHL_ipsi_: 9.1). At midline, CHL_contra_ neurons had poorer JNDs at midline compared to CHL_ipsi_ and Control neurons (median ± SD: Control: 7.8 ± 164.4 dB ILD; CHL_contra_: 15.8 ± 396.1 dB ILD; CHL_ipsi_: 8.2 ± 73.8 dB ILD).

In summary, we find that after a transient developmental CHL in one ear, ILD-sensitive neurons in the auditory midbrain display a reduction in sensitivity to ILD compared to littermate Controls. Further, these animals displayed altered binaural processing in the auditory brainstem along with deficits in spatial discrimination of sounds that largely contributes to ILD cues (i.e., high-frequencies).

### Early unilateral CHL does not alter monaural response properties in the auditory midbrain

One potential explanation for altered ILD processing by IC neurons is that monaural response properties may be compromised following Early CHL. Therefore, we examined monaural frequency tuning curves, rate level functions, and first spike latencies (see “Methods”) for all ILD-sensitive neurons.

S4A Fig shows an example “response area curve” (a 3D surface plot of frequency × intensity × firing rate) from which the neuron’s characteristic frequency (CF) can be determined (e.g., see asterisk). S4B Fig shows the neuron threshold as a function of unit CF for Control (*n* = 40) and Early CHL animals (CHL_contra_, *n* = 39; CHL_ipsi,_ *n* = 28). Histograms for CF and threshold were plotted on the *x*- and *y*-axes, respectively, to aid in visualization of data distribution. The mean neuron threshold (±SD) was 45.88 ± 13.4 dB for Controls, 52.4 ± 11.5 dB for Early CHL_contra_, and 49.82 ± 12.9 dB for Early CHL_ipsi_. A one-way ANOVA found no significant differences between the threshold means (*F*_2,104_ = 2.71, *p* = 0.071). The CFs between the groups displayed similar ranges in frequency (Controls: 0.86–25.73 kHz; CHL_contra_: 0.649–15.91 kHz; CHL_ipsi_: 0.559–26.86 kHz), although neurons ipsilateral to the CHL had a higher proportion of high CF units (>8 kHz). The range of CFs observed are comparable to the guinea pig behavioral audiogram reported in Heffner and colleagues [57].

Next, we examined the rate level functions (RLFs) from all neurons. Once the CF is determined for each unit (from the response area curve), the intensity at the contralateral ear (the excitatory ear) is varied to determine unit threshold and first spike latency to CF tone. S5A Fig shows an example RLF from an IC neuron (left panel). The right panel shows the associated spike rasters for each trial, at each sound intensity presented (30–90 dB at the contralateral ear). The dotted line indicates the onset of the spike responses (i.e., first spike latency). S5B Fig shows the first spike latency for Control and Early CHL neurons as a function of CF. The mean latency (±SD) for Controls is 17.28 ± 5.38 ms, for Early CHL_contra_ is 17.4 ± 3.54 ms, and for Early CHL_ipsi_ is 17.12 ± 6.92 ms. A one-way ANOVA revealed no significant differences between the first spike latency between Control and Early CHL neurons (*F*_2,104_ = 0.03, *p* = 0.98).

In summary, monaural response properties were not altered following developmental CHL as compared to Controls. Therefore, we attribute the altered ILD coding and poorer neural ILD JNDs following Early CHL to alterations to binaural, and not monaural, processing in IC neurons.

## Discussion

Experience-driven developmental plasticity of binaural processing has been widely examined at the level of the auditory thalamus [23] and cortex [18–22]. Fewer studies, however, have examined whether developmental auditory experience can also induce plasticity at earlier ascending nuclei, including the auditory brainstem, the initial site of synaptic convergence between input from both ears [30], or the auditory midbrain (inferior colliculus, IC) [22,24,26,82]. See [83] for review. Here, we report a prolonged maturation of the binaural auditory brainstem in guinea pigs (Figs 1 and 2), suggesting that binaural plasticity may be heightened, and therefore vulnerable to experience, during this time. Indeed, we find that developmental HL alters a brainstem readout of binaural function (Fig 4) which is not observed when the HL is induced in adulthood (Fig 5). Startle-based behavioral measures reveal poorer spatial resolution of sound sources, but only for high-frequency sound stimuli (Fig 6). Finally, single-unit recordings of auditory midbrain neurons reveal significantly poorer neural acuity to a sound location cue that largely depends on high-frequency sounds (Fig 7).

### Altered binaural processing in the auditory brainstem following developmental hearing loss

Following developmental CHL, we find alterations of the binaural interaction component (BIC) of the auditory brainstem response (ABR), including longer latencies (S2 Fig) and broader “tuning” to ITDs (Fig 4). Our findings are consistent with reports in both human and animal subjects. Laska and colleagues [66] found similar increases in BIC DN1 peak latency after rearing guinea pigs with a reversible CHL. They report latency differences of approximately 0.1–0.4 ms which is comparable to our study (approximately 0.13 ms). This study and others report longer latencies of later ABR waves that correspond to binaural auditory structures in children with a history of CHL [45,64,65].

An increase in BIC latency suggests that a developmental CHL can lead to temporal alterations in the binaural nuclei of the auditory brainstem. There are several potential contributions that may increase the latency of ABR waves, including increased conduction time between auditory nuclei in the ascending pathway or reduced temporal coding (i.e., synchronicity of neurons to stimulus). Myelination of axons, which contributes to conduction velocity, is an activity-dependent process [84]. In fact, acoustic trauma can result in demyelination of the auditory nerve resulting in slower conduction velocities [85]. Given that precise timing of axonal inputs is critical for brainstem processing of acoustical cues [77,86,87], asymmetrical CHL in the developing brainstem may lead to axon conduction imbalances between synaptic inputs from the left and right ear.

The BIC is strongly modulated by acoustical cues to sound location, including ITDs. In the present study, we find developmental CHL leads to decreases in the BIC amplitude at small ITDs (Fig 4C), reduced modulation of BIC amplitude to ITD (Fig 4D), and broader BIC versus ITD tuning functions (Fig 4E). The decrease in BIC amplitude suggests that there is less inhibition when comparing the binaural-evoked ABR with the sum of the monaural ABRs (the BIC is a negative peak, so the greater the inhibitory drive, the smaller the binaurally-evoked ABR, the larger the BIC peak). This suggests a CHL-induced disruption of the developing inhibitory binaural circuits. A reduction in BIC amplitude could also suggest less synchronous activity by neurons in the circuit. Broader BIC versus ITD curve widths could indicate that neurons in the E/I circuit are not as precisely tuned, requiring larger changes in ITD to synchronously modulate the BIC amplitude. Animals with asymmetrical acoustic experience during development could result in imbalances in excitation and inhibition. In fact, Clarkson and colleagues [29] found that a monaural CHL in young adult rats led to an upregulation of AMPA glutamate receptor subunits (GluA3) on bushy cells of the cochlear nucleus, and AMPA receptors mediate fast synaptic transmission in the auditory pathway. In the cochlear nucleus, inhibition is mediated by the activation of glycine receptors [88], which are reported to be downregulated in the cochlear nucleus following CHL [89]. Up- or down-regulation of excitatory and inhibitory neurotransmitter receptors reflects compensatory homeostatic mechanisms in neuronal circuits following external disruptions [90] and can alter the efficiency of synaptic transmission [91]. This aligns with prior work [82] showing ILD curve shifts in juvenile animals consistent with compensatory changes in excitation and inhibition following unilateral CHL. Additionally, other findings suggest that earplugging in adult animals can lead to demyelination in bushy cell axons of the brainstem trapezoid body [92], potentially resulting in conduction delays that could contribute to the subtle shifts in BIC-ITD curves that are observed following Adult-onset CHL (Fig 5F).

The developing auditory brainstem, including the LSO, is highly influenced by activity-driven modifications after the onset of hearing [35,93]. Indeed, we find that the BIC responses in newborn guinea pigs are poorly tuned to ITD and undergo a period of prolonged refinement (Fig 2F, 2H, and 2M). Further, the time-course of BIC tuning aligns with the age at which acoustical cues to sound location reach adult-like values (approximately P56) [59]. Given that auditory neurons in the brainstem must maintain rapid transmission and temporal fidelity [94], diminished acoustic experience in one ear could disrupt this maturation process leading to changes in the E/I balance in the brainstem. It should be noted that in adult mammals, across a variety of species tested (mice, rats, non-human primates, humans), the function relating the amplitude of the BIC to changes in ITD are statistically the same even though the size of the head in these species differs by nearly a factor of 10 [48,95]. This occurs because the BIC originates from the LSO and the tuning to ITD reflects synaptic interaction of excitation and inhibition, which is conserved across mammals.

### Spatial hearing deficits following developmental hearing loss

Human auditory perceptual skills mature over a long time, such as frequency resolution which matures by 6 months of age [96], to frequency discrimination, maturing as late as approximately 10 years of age for low-frequency sounds [97]. Intensity discrimination (i.e., distinguishing whether one sound is louder than the other) does not mature until after 10 years of age [98]. Infants can detect differences in intensity between two sounds of about 6 dB, and this decreases to about 2 dB by 4 years of age [99]. By comparison, adults can discriminate intensity differences of high frequency tones as little as 0.5 dB [100]. Children that experience CHL during these developmental ages often have long lasting binaural hearing deficits that can persist for years beyond resolution of the CHL [40,101–106].

Animal studies also report persistent perceptual deficits following developmental hearing loss. In a classic series of experiments, barn owls were subjected to unilateral CHL (i.e., earplug in one ear) either during development or as adults. When tested in an auditory orienting (head turn) paradigm after plug removal, both groups initially mislocalized the source in the direction of the previously occluded ear. However, the adult-CHL animals adapted to the corrected binaural inputs, while the developmental-CHL animals continued to mislocalize the source [107,108]. Similar observations have been reported for localization and tone-in-noise detection tasks in ferrets [19,109]. This likely reflects differential mechanisms of plasticity underlying adult versus developmental deprivation. Together, these studies suggest the existence of a binaural auditory “sensitive period” [107,108], beyond which amelioration of peripheral inputs may be ineffective in correcting aberrant central mapping.

Here, we find that Early CHL animals required larger spatial separations in the location of high-pass noise to detect a change in source location (Fig 6F and 6H). Broadband and high-pass stimuli conditions correspond to the relevant acoustical cues available to the guinea pig: broadband stimuli include both ITDs and ILDs, whereas high-pass stimuli provide access to primarily ILDs and spectral cues. For broadband stimuli, both groups showed significant prepulse inhibition (PPI) at all angles tested, consistent with a group-level MAA of ≤7.5°. For high-pass stimuli, however, Control animals showed significant PPI for angles ≥15°, whereas Early CHL animals showed significant PPI only for angles ≥45°, resulting in a group-level MAA estimate of 30–45°. The shift in the angle at which significant PPI emerges suggests a reduction in spatial resolution following Early CHL. To estimate the deficit in behavioral ILD sensitivity, acoustic transfer function measurements reported for guinea pigs were used [60]. Here, the average ILD versus azimuth slope (dB/degree) can be computed for sources ±30° about the midline based on a population of adult animals. For frequencies greater than 4 kHz (the frequency cutoff for our high-pass noise stimuli), the average ILD slope is approximately 0.2 dB/°. Using the high-pass noise behavioral discrimination thresholds (MAAs), the MAA can be multiplied by the average ILD slope to get an estimation of behavioral ILD sensitivity. The MAA for Controls was between 7.5–15°, so the estimated ILD sensitivity is 7.5–15° × 0.2 dB/° = 1.5–3 dB ILD. The MAA for Early CHL animals was between 30° and 45°, so the estimated ILD sensitivity is 30–45° × 0.2 dB/° = 6–9 dB ILD. Thus, we estimate that CHL we induced in developing guinea pigs impaired ILD sensitivity by approximately 4.5–6 dB. We interpret the PPI versus angle function in Early CHL animals as reflecting a rightward shift (Fig 6H), indicating that larger angular separations are required to elicit a detectable response (i.e., a shift in behavioral threshold). An alternative interpretation is that Early CHL animals exhibit a general reduction in PPI magnitude across all angles, resulting in a downward shift of the function. While we observed a small facilitation trend at small angles for right-to-left swaps in Early CHL animals (Fig 6F), this was not statistically reliable. Given that our behavioral threshold is defined as the smallest angle at which PPI is significantly different from 0, the observed pattern is most consistent with a lateral shift in spatial acuity threshold rather than a uniform attenuation or facilitation of the startle response Finally, this behavioral deficit is paralleled by differences in the latency and amplitude of the BIC DN1 peak of the ABR (Fig 4), and is consistent with prior human work showing that BIC properties correlate with spatial hearing performance [46,110,111].

### ILD-sensitive midbrain neurons from Early CHL carry less information regarding ILD, leading to impairments in ILD discrimination

The BIC and the behavioral results indicate that there are alterations in underlying binaural processing of ILD cues at the earliest levels of the auditory system. The initial processing of ILD cues occurs in the lateral superior olive (LSO) [33,112,113]. The LSO projects to the central nucleus of the inferior colliculus (IC), a crucial center for spatial hearing. Lesions of the IC result in severe deficits in localization tasks, but not necessarily in other basic auditory tasks [114,115]. The effect of altered inputs to the IC may also be manifested in clinical studies of children with histories of early CHL, who often exhibit altered BICs of the ABR that are predictive of impaired spatial hearing [45]. The latencies of the BIC indicate a brainstem-level deficit in binaural processing occurring by the level of the IC [48,50]. Given the difficulty of directly recording from ILD-sensitive cells in the LSO (i.e., the neural generator of the BIC (see [32] for review), we opted to record from neurons in the IC which receives direct LSO inputs [116]. Furthermore, the IC is required for the PPI of the acoustic startle [73], implying that any disruption in encoding of the cues to location by neurons in the IC would also disrupt the PPI of the acoustic startle to changes in sound location.

Unilateral CHL during development can lead to extensive changes to auditory brainstem anatomy [117,118] and physiology [22,24,26,82,119,120]. The neurophysiological changes that are observed in many of these studies suggest an imbalance of converging ipsilateral and contralateral inputs to the IC. For example, experiments conducted by Silverman and Clopton [26] showed that rearing rats with a unilateral CHL resulted in substantially reduced effective inhibitory input to the IC ipsilateral to the occlusion, and a considerable increase in effective inhibitory input to neurons in the contralateral IC. Similar results have been reported in the IC and cortex of the rat [22] and mouse [21]. Opposite results (increased ipsilateral inhibition, decreased contralateral inhibition) have been reported in the cat [120], barn owl [25] and chinchilla [82], perhaps indicative of species differences. All studies, however, demonstrated that CHL led to disruptions in inhibition and impaired neural ILD sensitivity that persist beyond resolution of the CHL.

Here, we observed differences in ILD sensitivity between IC neurons from Control and Early CHL animals (Figs 7 and S3). We found a reduction in maximum firing rate between Control and Early CHL_contra_ groups (IC recording contralateral to CHL side; S3C Fig), suggesting that the excitatory drive to the ear contralateral to the previous CHL is lessened. While there were no significant differences in the minimum firing rates S3D Fig, a proportion of neurons ipsilateral to the CHL (which largely provides the inhibitory drive to the IC) appear to have higher minimum firing rates, suggesting a lessened inhibitory input. For the 50% inflection point (or the half-maximum value) there were no significant differences between groups (S3E Fig), although there appears to be a “shift” in the inflection point towards negative ILD values indicating that a proportion of neurons is now encoding a different range of ILDs. For the ILD dynamic range, there is a significant increase in the dynamic range for neurons ipsilateral to CHL compared to Controls, and a similar trend for neurons contralateral to CHL (S3F Fig). There appears to be less effective inhibitory influences from the ear ipsilateral to the CHL resulting in neurons encoding larger ranges in ILD compared to Control neurons, which are encoding a narrower range of ILDs. For the ILD slope, the smaller values for CHL neurons indicates that the slope of the function is much shallower compared to Control neurons (S3G Fig). All else being equal, a shallowing of the rate slope indicates that the neurons carry less information about ILD [82]. Overall, the results are suggestive of an alteration in the excitatory/inhibitory input to the IC in Early CHL animals, leading to possible reductions in ILD sensitivity. Further, our earplug manipulation provided greater attenuation at higher frequencies (≥4 kHz; Fig 3B), suggesting a greater effects on acoustical ILD cues rather than ITD cues.

We opted to use a more quantitative approach to assess the impacts of Early CHL on neural coding: the mathematical framework of Fisher information (FI). FI quantifies how well a neuron can discriminate two stimulus values (e.g., two ILDs), thus measuring stimulus precision. FI is a useful metric that allows for the quantification of neural ILD discrimination− which, importantly, is a more direct comparison between neurophysiological responses and behavioral performance as they both measure discrimination of sound source locations. There were reductions in FI between Early CHL and Control neurons with an approximately 53% reduction for neurons contralateral to the CHL and an approximately 73% reduction for neurons ipsilateral to the CHL (Fig 7G and 7H). Using FI, it was determined how well neurons could discriminate changes in ILD (i.e., neural ILD JND): Early CHL neurons could discriminate changes of approximately 4–5 dB ILD which is twice that of Control neurons (ILD JND: approximately 2.6 dB; Fig 7I and 7J). Comparing the neural ILD JNDs, Early CHL impaired ILD sensitivity of ICC neurons by approximately 1.4–2.4 dB. The ILD discrimination thresholds obtained from the behavior and physiology both show similar impacts of Early CHL. For Control animals, the behavioral ILD threshold was estimated to be 1.5–3 dB ILD, while the average ILD JND for single neurons was approximately 2.6 dB ILD. The JNDs from Early CHL animals were also consistent, with a behavioral ILD JND estimated between 6 and 9 dB ILD and a neural ILD JND of approximately 4–5 dB ILD. Thus, both behavioral and physiological measures of ILD JNDs show at least a 2- to 3-fold increase following a developmental CHL compared to Controls.

## Conclusions

While mammalian studies of unilateral CHL have demonstrated plasticity of binaural function in the auditory cortex, thalamus and midbrain, it remains unclear whether unilateral deprivation also induces plasticity in the auditory brainstem. Foundational work in the barn owl has shown that developmental manipulations can profoundly alter binaural tuning in brainstem circuits; however, how these findings translate to mammals remains less clear. Furthermore, the relationship between developmental CHL-induced neural changes and spatial hearing outcomes is uncertain. Here, we report a prolonged maturation of the binaural auditory brainstem in the guinea pig by tracking auditory evoked potentials across development. Using this age range, we induced a reversible unilateral CHL and asked whether behavioral and neural maturation were disrupted. We found that developmental CHL altered a brainstem readout of binaural function which was not observed when the CHL was induced in adulthood. Startle-based behavioral measures revealed poorer spatial resolution of sound sources, but only for high-frequency sound stimuli. Finally, single-unit recordings of auditory midbrain neurons revealed significantly poorer neural acuity to a sound location cue that largely depends on high-frequency sounds. Taken together, these findings show that unilateral deprivation can disrupt developing auditory circuits that integrate binaural information and may give rise to the lingering spatial hearing deficits observed in human and animal studies.

## Methods

### Experimental subjects

A total of 85 guinea pigs (*Cavia porcellus*) (44 females, 41 males) were used in the study. Animals were used either for the Developmental ABR/BIC experiments or the conductive hearing loss (CHL) experiments. All pups were from our in-house breeding colony. Animals were housed on a 12-hr light/12-hr dark cycle with full access to food and water. All surgical and experimental procedures complied with the guidelines of the University of Colorado Anschutz Medical Campus Institutional Animal Care and Use Committee (IACUC) under protocol #00232 and the National Institutes of Health.

### Developmental ABR/BIC experiment

#### Animal preparation.

A total of 18 pigmented guinea pigs from an in-house breeding colony were used for ABR assessments across development (9 females, 9 males). Seven animals were used to track ABRs from birth (P1) through adult ages (>P56). The remaining 11 animals had 1–2 ABR measurements at different developmental ages. Prior to testing, animals were anesthetized intraperitoneally using a mixture of ketamine hydrochloride (<P14: 65–70 mg/kg; >P14: 80 mg/kg) and xylazine hydrochloride (<P14: 7 mg/kg; >P14: 8 mg/kg). The core body temperature was maintained by an electronically controlled heating blanket. Heart rate, respiration rate, and blood-oxygen levels (SpO_2_) were monitored throughout each experiment.

#### Experimental setup.

The experimental details are briefly described here as a more in-depth description can be found in Ferber and colleagues [62]. Stimuli were presented through custom stainless-steel insert earpieces using TDT System 3 (Tucker Davis Technologies, Alachua, FL) CF1 speakers powered by a TDT SA1 amplifier. Sound level and phase were calibrated for each session via Etymotic Research (Elk Grove Village, IL) ER-7C microphones, with probe tubes positioned approximately 2 mm into the ear canal. The calibration was applied using a 129-tap minimum phase filter, which ensures delivery of a temporally discrete click (see Beutelmann and colleagues 2015 [121]  for more details). The microphones were kept in place during the experiment, and their output was recorded simultaneously with the ABR signal to ensure signal fidelity throughout the duration of each experiment. The absolute sound pressure level was referenced to a 1-kHz tone. Recordings were made with platinum subdermal needle electrodes (F-E2-12 electrodes; Grass Technologies, West Warwick, RI) at the apex (active) and nape of the neck (reference) with a hind leg ground (Fig 2B). This configuration allows for comparison of monaural and binaural ABRs [62]. Before acquisition, sound-evoked ABR signals were preamplified (10,000×) and band-pass filtered (cutoffs 100 Hz and 3 kHz) by a WPI ISO-80 amplifier. An automated artifact rejection threshold, typically approximately 15 µV, was set before each recording session.

#### Recording parameters and data acquisition.

Click-evoked monaural ABR thresholds in each animal were assessed using clicks at increasing intensity (generally 20–90 dB SPL in 5 dB steps; click rate: 33/s). Click stimuli were used as it is a steep transient with a broad spectrum, activating a wide range of frequencies within a short time window [122]. Five hundred artifact-free repetitions were presented for each condition in randomized order. Monaural- and binaural- presented clicks were then varied across an ITD range spanning ±2,000 μs (±2,000, 1,000, 750, 500, 375, 250, 125, 0 μs; all conditions randomized), bandpass-filtered (100 Hz–3 kHz), and averaged. Clicks were presented at a fixed level that is ≥40 dB above the click threshold (80 dB). The range of ITDs presented here encompasses and exceeds the physiological range of ITD in guinea pig, which is approximately ±320 μs [60]. One thousand artifact-free repetitions were recorded for each condition. Presentation of stimuli and acquisition of evoked potentials were facilitated by a Hamerfall Multiface II (RME) sound card controlled by PC running custom MATLAB software in a Linux-based environment (OpenSUSE 13.1).

#### BIC computation, normalization, and Gaussian fitting.

Binaural and summed ABR waves were identiﬁed as local maxima and BIC waves as local minima by a custom MATLAB procedure. The BIC waveform was calculated for each ITD (BIC = ABR_Binaural_ − [ABR_LeftMonaural_ + ABR_RightMonaural_]) with the left ear and right ear ABR traces time-shifted before summation according to the timing of clicks corresponding to the ITD (see Fig 2B–2D). Normalization of BIC amplitude was applied to account for across-animal signal-to-noise variability in individual recordings [62]. Here, the BIC amplitudes were normalized to the average root mean square (RMS) of the two monaural ABRs. BIC peak amplitude versus ITD data are then fit with a four-parameter Gaussian curve: BIC_model_ (ITD) = B + A * exp(−0.5 * ((ITD − C)/*σ*)^2^) where *C* is the ITD of maximal BIC (i.e., curve center), *σ* is the curve width (e.g., standard deviation of distribution) of the Gaussian function, A is the modulation of the curve (e.g., amplitude), and B is the baseline BIC amplitude value (Fig 2G). For group averages, the BIC peak amplitudes were averaged at each ITD, then Gaussian fits were applied (Figs 4C and 5B). For analysis of each parameter, Gaussian fits were applied to BIC versus ITDs from individual sessions/conditions (Figs 4C inset and 5B inset).

A Spearman’s rho correlation analysis was used to assess whether there were significant developmental changes in the data (e.g., in amplitude, latency, Gaussian fit parameters). The Spearman’s correlation coefficient, *r*_*s*_, measures the strength and direction of association between two ranked variables, such as between the parameter of interest (e.g., BIC amplitude) and age. When a significant correlation is found, an exponential decay function is fit to the data. The methods used here are derived from the appendix of Eggermont and Moore [123] which describe the use of exponential functions to model maturational changes.

#### Excitatory–inhibitory model of the BIC.

Ungan and colleagues [51] proposed a computational model to explain the dependency of the BIC latency and amplitude with ITD by modeling the binaural interaction found in cells of the LSO, with contralateral inhibitory and ipsilateral excitatory inputs (IE cells). The Ungan model has 4 parameters, including the difference between the mean arrival times of the excitatory and inhibitory input to the binaural LSO cell, the standard deviations of the excitatory and inhibitory arrival times, and the duration of inhibition of the LSO cell. Later, Riedel and Kollmeier [124] adjusted the model to include an amplitude scaling factor (i.e., the four parameters are optimized by means of a chi-squared fit of the average BIC amplitude and latency data over subjects). This model was further refined in Benichoux and colleagues [48], which is the model presented below.

Here, we sought to simplify this model by reducing the number of parameters from 4 to 2 as we are not interested in the latency of DN1, but rather how the amplitude changes with ITD. Consider that spikes arrive at different times for the excitatory and inhibitory pathways:


$$E\sim N(\tau_{e},\sigma_{e}^{2})$$

$$I\sim N(\tau_{i},\sigma_{i}^{2})$$

Where *N*(*μ,σ*^2^) represents a normal (Gaussian) distribution with mean *μ* (the expected arrival time of spikes) and variance *σ*^2^ (the variability in spike timing).

The binaural difference (i.e., the BIC) is assumed to measure the number of excitatory spikes that are cancelled by inhibition. This is obtained when excitation occurs during inhibition, within a given duration *w* (the inhibition *window*). Therefore, the event *R* occurring when a spike is cancelled by inhibition is defined as a function of the arrival times *I* and *E*, by:


R=[I<E<I+w]=[0<E−I<w]

Where [*x*] is 1 if *x* is true and zero otherwise. That is, *x* is an indicator function that equals 1 if excitation occurs within the inhibition window following inhibition and 0 otherwise. Because the two random variables *E* and *I* are independent, normally distributed random variables, we know that the distribution of U=E−I is also normal:


$$E-I=U\;\sim \;N\left({\tau_{e}-\tau }_{i},\sigma_{e}^{2}+\sigma_{i}^{2}\right)$$

Since $\sigma_{e}^{2}$ and $\sigma_{i}^{2}$ play an interchangeable role, we let $\sigma^{2}=\sigma_{i}^{2}+\sigma_{e}^{2}$, and for similar reasons, only the difference in mean arrival times plays a role, so we let ${\tau =\tau_{e}-\tau }_{i}$. In turn, the probability that *R* is one is described with:


[R=1]=[0<U<w]

With $U\;\sim \;N\left(\tau ,\sigma^{2}\right)$. This probability is obtained with the cumulative function of a normal random variable. The probability that a spike gets canceled by inhibition is thus given by:


$$P[R=1]=\int_{0<u<w}\;f_{U}(u)du=\frac{1}{2}\left(1+\left(\frac{w-\tau }{\sqrt{2}\sigma }\right)\;\right)-\frac{1}{2}\left(1+\left(\frac{-\tau }{\sqrt{2}\sigma }\right)\;\right)$$

Where $f_{U}(u)$ is the probability density function of U. We can then identify the frequency of R=1 to the magnitude of the BIC. Indeed, consider that the response of the population of LSO neurons is the summed response of N neurons: the mean rate $r=\frac{1}{N}\sum_{i}\;\left[R_{i}=1\right]$. If we assume that all $R_{i}$ are independent and identically distributed, the response of the population is given by the frequency of R=1, as given by the above equation. In order to obtain a model of the amplitude of the BIC, we have to consider LSO populations on both sides. We note that, on the left LSO, $\tau_{e}=-ITD/2$ and $\tau_{i}=ITD/2$ while on the right LSO, $\tau_{e}=ITD/2$ and $\tau_{i}=-ITD/2.$ We are left to consider two normal random variables:


$$U_{L}\;\sim \;N\left(-ITD,\sigma^{2}\right)$$

$$U_{R}\;\sim \;N\left(ITD,\sigma^{2}\right)$$

Whose responses follow from the previous equations:


$$r_{L}(ITD)=\frac{1}{2}\left(1+\left(\frac{w-ITD/2}{\sqrt{2}\sigma }\right)\;\right)-\frac{1}{2}\left(1+\left(\frac{-ITD/2}{\sqrt{2}\sigma }\right)\;\right)$$

$$r_{R}(ITD)=\frac{1}{2}\left(1+\left(\frac{w+ITD/2}{\sqrt{2}\sigma }\right)\;\right)-\frac{1}{2}\left(1+\left(\frac{ITD/2}{\sqrt{2}\sigma }\right)\;\right)$$

And finally:


$$r(ITD)=r_{L}(ITD)+r_{R}(ITD)$$

This simple expression provides us with a model of the BIC that depends on two parameters:

(1) σ representing the precision of arrival times of excitatory and inhibitory inputs to LSO cells
(2) w representing the temporal duration of the inhibition

The model predicts that the BIC should be roughly bell-shaped with a peak at 0 ITD. The width of the bell and its maximum depend on the relative values of ITDs, *σ*, and *w*. This model can then fit the BIC amplitude versus the ITD data. To do this, avoiding superfluous free parameters, the BIC amplitudes must be normalized between 0 and 1 using the following expression:


$$BIC(ITD)=\frac{BIC(ITD)-BIC_{baseline}}{BIC(0)-BIC_{baseline}}$$

Where BIC(0) is the BIC at 0 ITD, and we obtain the baseline BIC using the BIC at very large ITDs (higher than 2 ms):


$$BIC_{baseline}=\;<BIC(|ITD|>2)>$$

A least-squared fit can then be applied to the BIC amplitude values.

### Conductive hearing loss (CHL) experiments

#### Animal preparation.

A total of 58 pigmented guinea pigs were used for CHL experiments. For developmental CHL, newborn animals from our breeding colony were divided into two groups shortly after birth (P0–P1): animals raised with normal hearing (Controls, *n* = 25; 11 males, 14 females) and animals raised with a unilateral CHL (designated as “Early CHL” throughout the paper; *n* = 29; 11 males, 18 females). An additional 9 normal-hearing adults were used for physiology (8 males, 1 female) and were included in the Control physiology dataset. Animals were raised until adulthood, defined here as when the head and pinnae and hence the binaural acoustic cues to location reach adult dimensions, which occurs at postnatal day 56 (P56; i.e., 8 weeks after birth) [59], after which the earplug, if present, was removed. The following were then assessed: (1) auditory brainstem responses (ABRs), (2) spatial discrimination via a startle-based approach, and (3) physiological recordings in the inferior colliculus. All testing was completed within 1 week of plug removal; see Fig 3C for a timeline of experiments.

A smaller group of adult guinea pigs (>P56; *n* = 4) from our in-house colony were used to evaluate impacts of unilateral CHL in adulthood. For these experiments, baseline ABR assessments were completed (designated as “Pre-CHL”; 3 assessments per animal). after which a unilateral CHL of the same duration as that employed for the “Early CHL” group (8 weeks) was induced via earplugging. Following earplug removal, ABRs were collected for the post-CHL assessments (designated as “Post-CHL”; 2 sessions per animal; see Fig 5A for a timeline of experiments).

Prior to physiological testing (ABRs for Early CHL and Adult CHL groups; single-unit recordings for Early CHL groups), animals were anesthetized with an intraperitoneal injection of ketamine hydrochloride (80 mg/kg) and xylazine hydrochloride (8 mg/kg). Subsequent maintenance doses were administered when needed (60–70 mg/kg ketamine every hour, 4 mg/kg xylazine every other hour). The core body temperature was maintained by an electronically controlled heating blanket. Heart rate, respiration rate, and blood–oxygen levels (SpO_2_) were monitored throughout each experiment.

#### Earplug method of inducing CHL.

Custom silicone earplugs (Microsonic Ready-press ear mold impression system; Westone Laboratories Inc, Colorado Springs, CO) were used to induce a transient, reversible conductive hearing loss (CHL). Prior to insertion of silicone material, the ear canal was cleaned with Epi-Otic Advanced Ear Cleaner to prevent infection. Plugs were placed in one ear (left) within one day of birth (P0–P1) or in adulthood (>P56) and sealed with 3 M Vetbond Tissue Adhesive. Care was taken to ensure that the silicone material did not go near the tympanic membrane, which can cause irritation and discomfort (see Fig 3A for approximate depth of plug within the ear canal). Plugs remained in place for 8 weeks, until developing animals reached adulthood (P56). We defined adulthood as the age at which the guinea pig head and external ear dimensions, which give rise to the binaural cues to sound location (ILD, ITDs), reach adult ranges [59]. Adult-onset CHL animals had earplugs in place for the same duration (8 weeks). Earplugs were checked 1–2× daily and replaced as needed.

Earplug attenuation was determined by placing a microphone (Type 4182, Bruel and Kjaer) deep within the ear canal of cadavers (similar to the methods described in [60]). A small puncture was made behind the pinnae in the mastoid region and the probe tip was inserted through to the ear canal; this was done so that the microphone would not be obstructing the delivery of the sound stimuli or the placement of the plugs). A free-field speaker presented a sweep in tone frequencies, from 100 Hz to 30 kHz (100 Hz steps) and the resultant dB SPL measured by the microphone was obtained before and after placement of 4 different silicone plugs (*n* = 4 samples, Fig 3B). Silicone earplugs were found to provide a mild to moderate temporary hearing loss, with attenuation levels ranging from <10 dB SPL at frequencies less than 4 kHz (mean: 10 ± 7.5 dB SPL) to 10–35 dB SPL at frequencies greater than 4 kHz (mean: 23.7 ± 9.4 dB SPL; Fig 2B). The attenuation levels provided by the silicone plugs at frequencies above 1 kHz are consistent with those reported for foam earplugs in other species (barn owl [107]; chinchilla [125]; ferret [109]). However, at frequencies below 1 kHz, silicone earplugs provide less attenuation compared to foam plugs used in chinchillas (0–5 dB SPL versus 10–20 dB SPL) [125]. This is likely due to differences in earplug material, ear canal size, and volume of plug used.

#### Startle-based method for assessing spatial hearing.

Experiments were performed in a double-walled, sound-attenuating chamber (interior dimensions: approximately 3 × 3 × 3 m; Industrial Acoustics Company, IAC, Bronx, NY) lined with acoustical foam. The animal was placed in a custom-built acoustically transparent wire-mesh cage mounted on a polyvinyl chloride (PVC) post anchored to a flexible polycarbonate platform. The cage was oriented such that the animal faced forward towards the center loudspeaker (Fig 6A). Proper orientation of the animal was visually monitored using a closed-circuit infrared camera. Prepulse stimuli were presented from 25 identical loudspeakers (Morel MDT-20) spaced along a 1 m radius semicircular hoop at 7.5° increments, from −90° (right) to +90° (left). The array of speakers was oriented horizontally (i.e., 0° elevation) for all experiments. An additional loudspeaker (Faital Pro HF102) positioned approximately 25 cm above the animal provided the startle-eliciting (ES) stimuli (20 ms duration broadband noise bursts presented at 110–120 dB SPL). The startle response of the animal was captured using a cage-mounted accelerometer (Analog Devices ADXL335). Generation of the sound stimuli and recording of the startle signal was controlled by custom written MATLAB (MathWorks) software controlling three Tucker-Davis Technologies (TDT) Real-time Processors (RP2.1). The startle response amplitude was calculated as the RMS of the accelerometer output in the first 100 ms period after the delivery of the ES.

Behavioral spatial acuity was assessed using the speaker-swap (SSwap) paradigm [70,71], which quantifies the smallest angle for which a change in speaker location can be detected by the animal (i.e., the minimum audible angle) [74]. Guinea pigs reflexively startle in response to loud, unexpected sounds (Fig 6B), but presentation of a detectable cue (the “prepulse”) prior to the loud sound reduces the startle response (prepulse inhibition, PPI; Fig 6C). Here, the prepulse signal is a swap of continuous noise (broadband or high-pass) presented from one speaker to a second speaker, prior to presentation of a startle stimulus (110–120 dB SPL; interstimulus interval, ISI, between prepulse and ES: 300 ms). An ISI of 300 ms was selected to avoid prepulse facilitation, which has been observed in guinea pigs at shorter intervals [70]. The duration of the stimulus prior to the speaker swap averaged 20 s and varied pseudorandomly from trial to trial between 15 s and 25 s in 1 s increments. Immediately after the speaker swap and delivery of the startle stimulus another initial sound source location was chosen pseudorandomly and the process repeated. The direction of the swap was also randomized (e.g., noise swaps from left-to-right or right-to-left speakers). Five angles of the speaker swap prepulse were tested (7.5°, 15°, 30°, 45°, 90°; Fig 6A). Testing was performed across 2 sessions over 2 days and was limited to <60 min per session to avoid habituation to the startle response [126].

Responses are the average of at least 10 repetitions and are shown in units of prepulse inhibition (PPI). PPI is defined as 1 minus the ratio of RMS amplitude of the prepulse condition (speaker swap present) divided by the control condition (no speaker swap):


$$\text{PPI}\;=\;\text{1}\;-\;[\text{StartleRespons}{\text{e}}_{\text{Prepulse}}/{\text{StartleResponse}}_{\text{Control}}]$$

A PPI of “1” indicates a complete suppression of startle (i.e., detection of the swap), a “0” indicates that the startle response for the swap condition is no different from the control response (i.e., no detection of swap), and “<0” indicates a facilitation, or enhancement, of the startle response. For each condition, there were an equal number of non-swap control conditions that were pseudorandomly distributed over the total number of trials. The minimum audible angle (MAA) was defined as the smallest speaker swap angle that the animals reliably detected, as indicated by a cross-trial averaged startle response significantly different from that observed in the absence of the prepulse (swap) stimulus. MAA was computed at the group level using data pooled across animals within each condition. Specifically, a repeated-measures analysis of variance (ANOVA) was used to determine whether there was a significant effect of speaker swap angle on PPI (random factor: subject; repeated measure: angle), followed by Bonferroni-corrected two-tailed Student’s *t*-tests to identify the smallest angle at which the group-level PPI was significantly different from 0.

#### Single-unit extracellular recordings in the auditory midbrain.

After completion of ABR and behavioral tests, anesthetized animals (≥P59) were placed in a double-walled sound attenuating chamber (IAC, Bronx, NY) for physiological testing. Following methods of Jones and colleagues [78], hair was removed from the ventral aspect of the neck and a tracheal cannula was implanted to monitor respiration and ensure ease of breathing. The animal was then placed on a custom bite bar fixed to a stereotaxic apparatus (model 1430, David Kopf Instruments). Hair was removed from the top of the head, and a midline incision was made to expose the skull and the bony ear canals. Custom stainless-steel earpieces were then secured into each ear canal. Closed-field speakers were attached to the left and right earpieces to present acoustic stimuli. Probe microphones (Type 4182, Bruel and Kjaer) incorporated in the insert earpieces were used to calibrate tones between 50 Hz and 20 kHz. An approximately 2−3 mm craniotomy was made near the midline of the skull (at the interaural axis) to expose cortex overlying the inferior colliculus (IC). For the Early CHL animals, recordings were made in the IC both contralateral and ipsilateral to the previously-plugged ear (Fig 7C). Parylene-coated Tungsten microelectrodes (2−5 MΩ; MicroProbe) were secured to a micromanipulator and were advanced remotely outside of the chamber while presenting search stimuli (repeating tone sweeps, from approximately 100 Hz to 20 kHz). The correct placement of the electrode is validated when neurons reliably respond to auditory stimuli. Neurons in the central nucleus of the inferior colliculus (ICC) were targeted for physiological assessments. The ICC is a tonotopically organized auditory midbrain region where neurons are responsive to low frequencies in the dorsal ICC (<3 kHz in the guinea pig) and higher frequencies in ventral ICC (>16 kHz) [127]. As the electrode is advanced ventrally, the frequency selectivity of ICC neurons increases (i.e., from approximately 100 Hz to approximately 20 kHz), a feature that is not observed in other IC regions [128]. Thus, we use this to verify the correct recording location in the central nucleus. Unit responses were amplified (ISO-80, WPI, Sarasota, FL; Stanford Research Systems, SRS 560, Sunnyvale, CA) and filtered (300−3,000 Hz). A BAK amplitude-time window discriminator (Model DDIS-1, Mount Airy, MD) was used to evaluate neuron responses and spike times were stored at a precision of 1 μs via a Tucker-Davis Technologies (TDT, Alachua, FL) RV8.

Units were selected for further study if the spike waveforms exhibited good signal-to-noise ratio, the responses were strong and non-adapting to auditory stimuli, and the responses exhibited dorso–ventral tonotopy. For each isolated ICC neuron, the characteristic frequency (CF), rate-level function at CF, and binaural sensitivity to interaural level differences (ILDs) was assessed. The CF for each isolated unit was estimated from obtaining a three-dimensional plot of signal frequency (kHz), signal intensity (dB), and firing rate (spikes/sec; S4A Fig). After determining the CF of an isolated unit, the threshold of the neuron was assessed by varying the intensity of a CF tone presented to the ear contralateral to the electrode (e.g., recording in the left IC, the intensity presented to the right ear would be varied) to get a rate-level function (S5A Fig). From the rate-level function, the contralateral signal intensity that elicited approximately 50% of the maximal firing rate (typically 50–60 dB SPL and approximately 20 dB above threshold) was documented and used for subsequent binaural testing.

Binaural sensitivity to ILDs was assessed by fixing the sound intensity of a 200-ms CF tone at the contralateral ear (the “excitatory” ear) at approximately 20 dB above threshold and varying the sound intensity presented at the ipsilateral ear (the “inhibitory” ear) across a ±30 dB range in 5 dB steps; Fig 7A–7B, allowing us to manipulate the level of inhibition with a constant level of excitation. For example, when recording in the left IC, if the threshold of an isolated unit was 40 dB SPL, then the level at the right ear was be fixed at 60 dB SPL and the intensity presented to the left ear varied from 30 to 90 dB. Each ILD cue was presented 20 times, with cue values presented in random order over the course of a testing block. Raw rate-ILD data were fit with a four-parameter sigmoidal logistic function of the form, *y*(*α*) = **A* *+ ((*D −* *A*)/(1 + *expB*(*α*-*C*))) where *α* is the ILD (ipsi SPL − contra SPL), *A* is the minimum firing rate, *D* is the maximum firing rate, *C* is the inflection point (referred to as the “50% inflection point”), and *B* is a width parameter, the sign of which also controls the direction of inflection. Units were classified as ILD-sensitive if the firing rate was modulated by at least 50% with varying sound intensity to the ipsilateral (inhibitory) ear and the *R*^2^ of the fit was at least 0.70. The fit parameters include the 50% inflection point (i.e., half-maximum ILD value, or the ILD yielding 50% of maximal firing), the rate-ILD slope (spikes/s/dB ILD, computed at half-max ILD), and the ILD dynamic range (the range of ILD producing 10%–90% of maximal firing; S3A Fig) [81,116]. A total of 107 isolated units were classified as ILD-sensitive according to these criteria, including *n* = 40 units from Control animals and *n* = 67 units from CHL animals (of these, 39 were recorded in the IC ipsilateral to the earplug, and 29 were recorded in the IC contralateral to the earplug.

The ILD coding acuity of ICC neurons was next assessed according to the Fisher information (FI) conveyed by fitted rate-ILD functions. FI can be used to quantify the precision with which a neuron’s responses discriminate adjacent stimulus values (e.g., two ILDs). Here, FI is expressed in physical units (1/dB^2^) which can be used to compute neural ILD discrimination (i.e., ILD just-noticeable-differences, JNDs), and allow for a comparison between neural and behavioral acuity of sound source locations. The FI computation used here is also described in detail elsewhere [75,77–79,81]. FI is computed as $FI(ILD)=\frac{{r^{\prime }(ILD)}^{2}}{{\sigma (ILD)}^{2}}$

Where *r′* (ILD) is the derivative of the spike count versus ILD function with respect to ILD, and *σ* (ILD)^2^ is the variance of spike count across ILD. Here, we assumed a Poisson distribution such that the numerical value of *σ* (ILD)^2^ was set to equal the numerical value of the spike count (i.e., the number of spikes expected for a 1 second stimulus). Since FI considers the slope and variance of spiking across stimulus values, it can easily be converted to a signal detection theoretic measure [77,80] by evaluating the change in stimulus value (ILD) necessary to achieve 1 standard separation in spiking distributions (i.e., the neural JND for a *d*′ of 1, which corresponds to 76% correct discrimination in a 2-alternative forced choice psychophysical task). This value is given by the following:


$$JND(ILD)=\;\frac{1}{\sqrt{FI(ILD)}}$$

where *JND(ILD)* gives the neuron’s ILD JND, and *FI(ILD)* is the FI as a function of ILD as computed in the *FI(ILD)* equation above. See Fig 7D for an overview of the FI computation pipeline.

#### Statistical tests.

Statistical tests were performed using a significance level of **p* *< 0.05 (*α *= 0.05), unless otherwise indicated. Note, no statistical comparisons were made between the Early CHL and Adult CHL groups for ABR measures, as these experiments differed in design (Early CHL: between-subjects; Adult CHL: within-subjects).

## Supporting information

S1 Fig Developmental CHL does not alter click-evoked monaural ABRs. **(a)** Example left monaural ABR trace for a littermate Control animal (#124111). Traces are in response to broadband click stimuli and are shown for different stimulus intensities (20–90 dB SPL in 5 dB steps). Traces shown are the average of 500 repetitions. The dotted vertical line indicates the stimulus onset of the click. ABR thresholds were measured by identifying the lowest stimulus level at which a response was visually detectable, regardless of the specific wave. For instance, the threshold for this example would be 40 dB SPL (black asterisk). (**b**) Click-ABR thresholds are not significantly elevated in animals raised with CHL (Mann–Whitney U, n.s.: *p* > 0.01). Circles depict identified thresholds from monaural ABRs of Control animals (black, left and right ABRs were combined) and animals raised with an earplug (red). For the Early CHL animals, data were split into two groups: thresholds ipsilateral (dark red) and contralateral (bright red) to the previously occluded ear. Boxplots indicating the median, 10th, 25th, 75th, and 90th percentiles with error bars are shown for each group. The median thresholds (±SD) for Controls, Early CHL_ipsi_, and Early CHL_contra_ are 35 ± 7.2 dB SPL, 40 ± 7.9 dB SPL, and 35 ± 5.8 dB SPL, respectively. (**c–e**) Latencies (ms) for monaural ABR waves I **(c)**, III **(d)**, and IV **(e)** for Control (black) and Early CHL animals (ipsi to CHL: dark red; contra to CHL: bright red). Wave II was not included as it is not always identifiable in the ABR traces. (**f–h**) Amplitudes (µV) for monaural ABR waves I (**f**), III (**g**), and IV (**h**) for Control and Early CHL animals. For (**c–h**), the top inset shows an example ABR with the wave of focus emphasized. Each symbol indicates measurements taken from the left and right ear ABRs; Controls include both the left and right (*n* = 38 traces), whereas the CHL groups are split into measurements ipsilateral and contralateral to the developmental CHL (*n* = 21 traces for both groups). Boxplots indicating the median, 10th, 25th, 75th, and 90th percentiles with error bars are shown for each group. n.s. (Mann–Whitney U): *p* > 0.05. The data underlying this figure can be found at 10.17605/OSF.IO/KAW34.
(EPS)

S2 Fig Early CHL alters BIC latency. Top panel: The latency (ms) of the BIC is defined as the time from stimulus onset to the time of the DN1 negative peak of the BIC. Bottom panel: BIC latency for Control (*n* = 19) and Early CHL (*n* = 21) animals. Circles indicate individual values and the boxplots show the group median and the 10th, 25th, 75th, and 90th percentiles along with the associated error bars. Early CHL animals exhibit longer BIC latency than their littermate controls (Control median ± SD: 3.25 ± 0.19 ms; Early CHL: 3.38 ± 0.18 ms; Mann–Whitney U, *p* = 0.03). The data underlying this figure can be found at 10.17605/OSF.IO/KAW34.
(EPS)

S3 Fig Rate-ILD functions for Control and Early CHL inferior colliculus (IC) neurons. **(a)** Example rate-ILD function for an ILD-sensitive IC neuron. A sigmoid function is fit to all data to compare parameters between groups. Neurons were considered ILD-sensitive when their discharge rates were modulated by ≥50% with increasing sound intensity to the ipsilateral ear (i.e., increasing the inhibitory drive). **(b)** Spike rasters for example ILD-sensitive unit shown in **(a)**. From the rate-ILD functions, maximum **(c)** and minimum **(d)** spike rates were plotted from Controls (blue) and Early CHL animals (contra to plug: bright red; ipsi to plug: dark red). Maximum spike rates from neurons contralateral to the previous CHL are significantly smaller than from Control neurons and neurons ipsilateral to previous CHL. There are no significant differences between the groups for minimum spike rates. **(e)** The 50% inflection point from the sigmoid fits were not significantly different between groups. **(f)** The ILD dynamic range for neurons ipsilateral to the previous CHL were significantly different from Controls, whereas they were not significant for neurons contralateral to the previous CHL. **(g)** The ILD slope was significantly smaller for both CHL groups compared to Controls. N.s.: *p* > 0.05; *: *p* < 0.05; **: *p* < 0.01; ***: *p* < 0.001. The data underlying this figure can be found at 10.17605/OSF.IO/KAW34.
(EPS)

S4 Fig Monaural IC tuning properties from Control and Early CHL animals. **(a)** Response area curves (RACs) were collected from all IC neurons to determine the characteristic frequency (CF) and the unit threshold. **(a)** Example RAC for an IC neuron. The asterisk (*) indicates the frequency at which the neuron is most sensitive to (CF: 4.7 kHz; threshold: 35 dB SPL). **(b)** Neuron threshold as a function of CF for Control (blue) and Early CHL animals (neurons contralateral to CHL, bright red; neurons ipsilateral to CHL, dark red). Only neurons that are sensitive to interaural level differences (ILD) are shown here (see “Methods” for inclusion criteria). Histograms for CF and threshold are plotted on the *x*- and *y*-axes, respectively, to show distribution of CF and thresholds for Control and Early CHL neurons. The data underlying this figure can be found at 10.17605/OSF.IO/KAW34.
(EPS)

S5 Fig Rate level functions and first spike latencies for IC neurons. **(a)** Rate level functions (RLFs) were collected from IC to assess unit thresholds and first spike latencies (i.e., monaural response properties). The left panel shows an example RLF for an IC neuron. Frequency tones at the CF of the unit are presented at varying intensities at the contralateral ear (e.g., recording in the left IC, present tones from the right ear speaker). The right panel shows the spike raster for each intensity as function of time for the example neuron. The dotted line indicates the first spike latency (ms). **(b)** First spike latency as a function of CF from Control (blue) and Early CHL animals (neurons contralateral to CHL, bright red; neurons ipsilateral to CHL, dark red). There are no significant differences in latencies between Control or Early CHL neurons (*F*_2,104_ = 0.03, *p* = 0.98).
(EPS)

## Abbreviations

ABR: auditory brainstem response
BIC: binaural interaction component
CHL: conductive hearing loss
ILD: interaural level difference
ITD: interaural time difference
LSO: lateral superior olive
MAA: minimum audible angle
MNTB: medial nucleus of the trapezoid body
PPI: prepulse inhibition
RLF: rate level function
